# Anion carriers as potential treatments for cystic fibrosis: transport in cystic fibrosis cells, and additivity to channel-targeting drugs†
†Electronic supplementary information (ESI) available: Experimental details and additional data for compound synthesis, binding studies, transport studies and biological data. See DOI: 10.1039/c9sc04242c


**DOI:** 10.1039/c9sc04242c

**Published:** 2019-10-02

**Authors:** Hongyu Li, Hennie Valkenier, Abigail G. Thorne, Christopher M. Dias, James A. Cooper, Marion Kieffer, Nathalie Busschaert, Philip A. Gale, David N. Sheppard, Anthony P. Davis

**Affiliations:** a School of Physiology , Pharmacology and Neuroscience , University of Bristol , Biomedical Sciences Building, University Walk , Bristol BS8 1TD , UK . Email: D.N.Sheppard@bristol.ac.uk; b School of Chemistry , University of Bristol , Cantock's Close , Bristol BS8 1TS , UK . Email: Anthony.Davis@bristol.ac.uk; c Chemistry , University of Southampton , Southampton SO17 1BJ , UK . Email: philip.gale@sydney.edu.au

## Abstract

Defective anion transport is a hallmark of the genetic disease cystic fibrosis (CF). One approach to restore anion transport to CF cells utilises alternative pathways for transmembrane anion transport, including artificial anion carriers (anionophores). Here, we screened 22 anionophores for biological activity using fluorescence emission from the halide-sensitive yellow fluorescent protein. Three compounds possessed anion transport activity similar to or greater than that of a bis-(*p*-nitrophenyl)ureidodecalin previously shown to have promising biological activity. Anion transport by these anionophores was concentration-dependent and persistent. All four anionophores mediated anion transport in CF cells, and their activity was additive to rescue of the predominant disease-causing variant F508del-CFTR using the clinically-licensed drugs lumacaftor and ivacaftor. Toxicity was variable but minimal at the lower end. The results provide further evidence that anionophores, by themselves or together with other treatments that restore anion transport, offer a potential therapeutic strategy for CF.

## Abstract 2

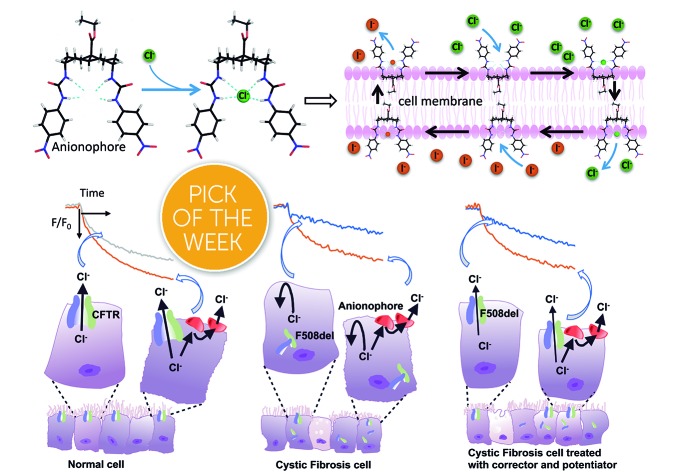
Synthetic anion transporters are active in cystic fibrosis cells, and are additive to clinically-approved drugs, suggesting new combination therapies for this lethal genetic condition.

## Introduction

Biologically-active synthetic transmembrane anion transporters (anionophores) have therapeutic potential in the genetic disease cystic fibrosis (CF).^1^–^5^ CF is caused by dysfunction of the anion channel cystic fibrosis transmembrane conductance regulator (CFTR), which plays a pivotal role in salt and water transport across epithelia.^6^–^8^ Although some drugs have been developed and clinically-licensed that target defects in CFTR, the large number of disease-causing variants and their rarity potentially limits the use of these agents.^9^–^12^ By contrast, anionophores might be developed to provide therapeutics for all individuals living with CF.^13^ They might be used alone or as part of a combination therapy with drugs that target faulty CFTR.

**Fig. 1:**
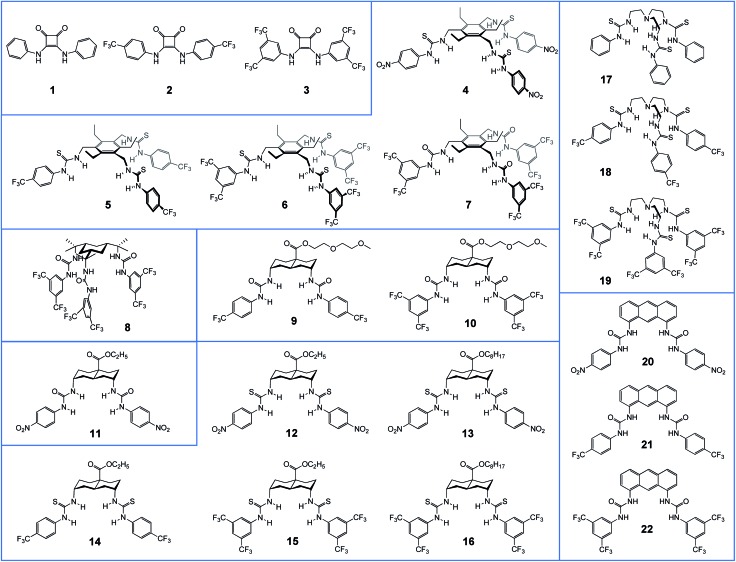
Structures of compounds **1–22** studied in this work.

We have developed a range of anionophores based on powerful anion binding sites created from hydrogen bond donor groups mounted on alicyclic scaffolds, which function as mobile anion carriers (Fig. 1).^14^–^16^ Scaffolds include steroids,^15^,^17^,^18^ *trans*-decalins,^19^,^20^ and substituted cyclohexanes,^21^ with the common design motif being the positioning of axially-directed H bond donors in 1,5 relationships. Conformational factors promote the convergence of NH groups to create binding sites with high affinities for inorganic anions.^14^,^18^,^21^ We have also developed alternatives, including squaramides,^22^ hexa-substituted benzenes,^23^ anthracenes^24^ and compounds based on the tris(2-aminoethyl)amine (tren) scaffold.^16^,^25^ While most studies of anionophores have used synthetic vesicles, we recently showed that some are also active in cells. An assay employing the genetically-encoded halide-sensitive fluorophore yellow fluorescent protein (YFP)^26^–^28^ gave encouraging results with several anionophores. In particular, the decalin-based bis-(*p*-nitrophenyl)-ureidodecalin **11** (Fig. 1) showed especially promising activity, including potency, persistence and a lack of toxic effects.^29^

The identification of optimal anionophores for CF treatment will require the screening of numerous candidates. The assay employed to identify **11** requires time-consuming fluorescence microscopy and is unsuitable for this purpose. Here, we report the implementation of a YFP-based assay using a standard plate reader. The assay was applied to a panel of **22** test compounds belonging to several compound classes, and was also used to extend the range of cell types studied. We identify three agents which show similar or improved performance relative to **11**, including one which is nearly twice as active. We find that these agents are potent and persistent, and provide further evidence that anionophore activity is possible without cytotoxicity. We also find that the anionophores are effective in CF cells, and demonstrate that their activity is additive to rescue of the predominant disease-causing variant, F508del-CFTR, using the clinically-licensed drugs lumacaftor and ivacaftor. Taken together, the data suggest that anionophores, either alone or together with CFTR modulators, are a potential therapeutic strategy for CF with wide utility.

## Results

### Anionophore synthesis and studies in synthetic membranes

Fig. 1 shows the anionophores investigated in this study, grouped according to structural similarity. We reported previously compounds **1–8**, **11**, **14–22** (for references, see Table 1). The new compounds **9**, **10** and **12**, **13** were prepared according to known methodology as discussed in the ESI.†

**Table 1:** Anionophore properties and binding/transport data

Compound			Binding		Transport in LUVs			Transport in YFP-FRT cells^*b*^	Reference
	MW (g mol^–1^)	clog *P*^*c*^	*K*_a_ to Cl^–^ in CHCl_3_^*d*^ (M^–1^)	*K*_a_ to Cl^–^ in DMSO^*e*^ (M^–1^)	Specific initial rate [*I*]^*f*^ (s^–1^)	Delivera-bility^*g*^	EC_50, 270 s_ (mol%) and *n*^*h*^	Corrected absolute initial slope |d*F*/d*T*| (×10^–3^)^*i*^ (s^–1^)	
**1**	264	2.6		2.6 × 10^2^			1.38 (1.7)	0	22
**2**	400	4.6		4.6 × 10^2^			0.06 (1.2)	0.3	22
**3**	536	6.6		6.4 × 10^2^	75^*b*^		0.01 (1.1)	0	22
**4**	790	7.2	n.d.^*j*^	3.8 × 10^2^	5			0.2	23
**5**	859	10.9	1.5 × 10^7^	3.0 × 10^2^	56	0.24^*b*^		0	23
**6**	1063	13.9	6.8 × 10^8^	4.5 × 10^2^	350	0.11^*b*^		0.2	23
**7**	1015	13.5	n.d.^*j*^	3.9 × 10^2^	50	0.18^*b*^		0.2	23
**8**	1021	13.9	n.d.^*j*^	3.9 × 10^2^	140	0.30^*b*^		0	21
**9** ^*a*^	689	6.6	1.4 × 10^8^	n.d.	9	1.55		1.1	
**10** ^*a*^	825	8.6	2.5 × 10^8^	n.d.	19	0.95		0.7	
**11**	569	4.5	n.d.^*j*^	6.8 × 10^2^	22	1.36		12.6	29
**12** ^*a*^	601	4.8	n.d.^*j*^	1.7 × 10^3^	310	1.12		17.2	
**13** ^*a*^	685	7.2	1.5 × 10^9^	n.d.	370	0.24		3.3	
**14**	647	7.3	1.2 × 10^8^	1.5 × 10^3^	200	2.02^*b*^		1.1	20
**15**	783	9.3	4.7 × 10^8^	2.4 × 10^3^	2600	0.41^*b*^		22.6	20
**16**	867	11.6	5.0 × 10^8^	2.6 × 10^3^	3800	0.03		0	20
**17**	552	4.2	5.2 × 10^5^^*b*^	1.8 × 10^3^			0.31 (1.9)	0.5	16 and 25
**18**	756	7.3	1.5 × 10^7^^*b*^	9.6 × 10^2^	36^*b*^		0.077 (4.8)	1.1	16 and 25
**19**	960	10.4	1.5 × 10^9^^*b*^	4.4 × 10^3^	590^*b*^	2.98^*b*^	0.042 (5.0)	12.2	16 and 25
**20**	537	6.9	n.d.^*j*^	2.6 × 10^3^	2100	0.82		8.4	24
**21**	583	9.3	n.d.^*j*^	2.2 × 10^3^	1200	0.62		1.7	24
**22**	719	11.4	n.d.^*j*^	3.0 × 10^3^	1900	0.45		9.0	24

^*a*^New compound.

^*b*^New data.

^*c*^Calculated using TorchV10lite.

^*d*^Obtained by extraction of Et_4_N^+^Cl^–^ from water into chloroform at 303 K.^31^

^*e*^Obtained from ^1^H NMR titrations with Bu_4_N^+^Cl^–^ in DMSO-d_6_/H_2_O (200 : 1) at 298 K.

^*f*^Transporter preincorporated in LUVs. Specific initial rate [*I*] = initial slope of *F*_0_/*F vs.* time *t*, divided by the transporter/lipid ratio in LUVs. LUVs (200 nm) are composed of 70% POPC + 30% cholesterol + transporter. Anion transport is induced by a [NaCl] gradient of 25 mM, with 225 mM NaNO_3_ inside and outside LUVs.

^*g*^Deliverability index (*D*) calculated by dividing *I* for the external addition of anionophore by that for preincorporated anionophore. Highly deliverable agents show values greater than 1, for reasons discussed in ref. 32.

^*h*^Concentration of externally added transporter (mol% carrier to lipid) for 50% Cl^–^ efflux in 270 s and Hill coefficient (*n*) during Cl^–^/NO_3_^–^ experiments, using LUVs (POPC; 200 nm) with 490 mM NaCl inside and 490 mM NaNO_3_ outside.

^*i*^Measurements from cells exposed to DMSO only was subtracted from that of DMSO (0.5% v/v) + transporter (50 μM) mixtures.

^*j*^Not determined due to low solubility in chloroform.

The four new anionophores were tested for affinity to Cl^–^ (in CHCl_3_ and DMSO, with tetra-alkylammonium counter-ions), and for anion transport (Cl^–^/NO_3_^–^ exchange) when preloaded into 200 nm large unilamellar vesicles (LUVs). The latter assay was performed using the well-established “lucigenin method”^30^ in which Cl^–^ influx into vesicles is followed through quenching of fluorescence from the halide-sensitive fluorophore lucigenin. The results are listed in Table 1, accompanied by corresponding values for the previously-reported compounds where available. Transport activities are given either as specific initial rates [*I*], a quantity defined in previous work to allow comparison of anionophores with widely differing activities,^20^ or as EC_50, 270 s_ values (see Table 1 footnote h).^16^Table 1 also lists parameters which are relevant to pharmaceutical potential (molecular weight and clog *P*) together with the results of a “deliverability” test applied to transporters **5–16** and **19–22**. In this test, anion transport by anionophores delivered externally to LUVs is compared with their action when preloaded into LUV membranes.^24^,^29^,^32^ Interestingly, Table 1 reveals that the new compound **12** has closely comparable properties to **11**, the decalin previously found to show promising biological activity,^29^ while promoting a considerably higher rate of transport in vesicles.

### Evaluation of anionophore activity in cells using a fluorescence-based assay

We recently demonstrated that anion transport by anionophores in individual cells can be monitored in real time using the genetically-encoded halide sensitive fluorophore YFP-H148Q/I152L.^26^–^29^ To efficiently identify compounds with improved biological activity, we have adapted our assay to investigate anionophore-mediated anion transport in a population of cells using Fischer rat thyroid (FRT) cells expressing YFP-H148Q/I152L (YFP-FRT cells) and a microplate reader.^27^ We treated YFP-FRT cells with anionophores (final concentration, 1–50 μM) or the vehicle DMSO (0.1–0.5% v/v) by direct addition to the phosphate-buffered saline (PBS) bathing the cells. After incubating YFP-FRT cells with anionophores for 10 minutes at 37 °C, we evaluated anion transport activity by adding I^–^ (100 mM), using a PBS iodide solution with Cl^–^ replaced by I^–^, and following cell fluorescence for 14 s. FRT cells are relatively impermeable to anions because they lack key ion channels found in epithelial cells, including CFTR.^33^ Thus, in the absence of anionophore, iodide cannot enter YFP-FRT cells and no quenching of fluorescence occurs. By contrast, in the presence of an anionophore, iodide influx occurs leading to fluorescence quenching.^29^

**Fig. 2:**
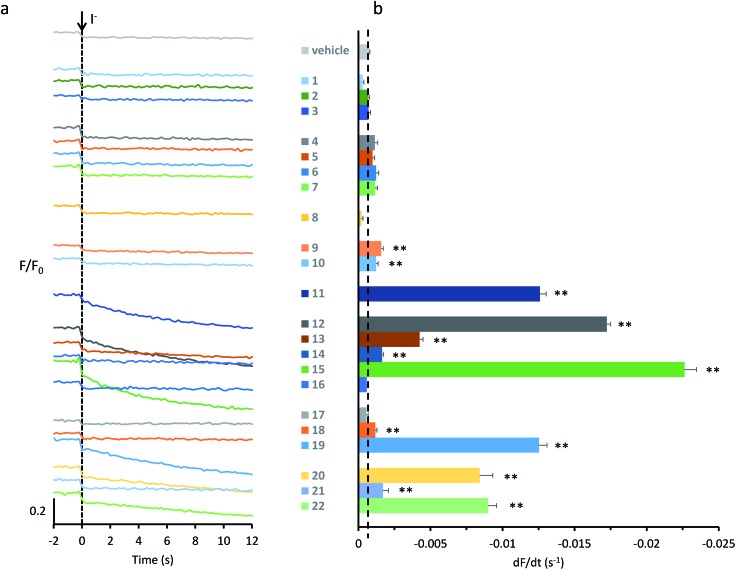
Screening anionophores for biological activity. (a) Representative time courses of cell fluorescence in YFP-FRT cells treated with the indicated anionophores (50 μM) or the vehicle DMSO (0.5% v/v) normalized to the fluorescence intensity before I^–^ (100 mM) addition at *t* = 0 s. (b) Anionophore-mediated anion transport in YFP-FRT cells determined from the initial slope of the fluorescence decay. The vertical dashed line indicates the initial slope of the fluorescence decay for control cells treated with DMSO (0.5% v/v). Data are means ± SEM (*n* = 16–64 from at least four independent experiments); **, *P* < 0.01 *vs.* DMSO.

Fig. 2a shows representative time courses for YFP-FRT cells treated with 22 test anionophores (50 μM) and the vehicle DMSO (0.5% v/v). When YFP-FRT cells were treated with DMSO, or if neither anionophore nor I^–^ were added, there was little or no change in the time course of fluorescence (Fig. 2a and ref. 29). Fig. 2a demonstrates that the previously-studied **11** generated a pronounced decay of fluorescence, recapitulating results using fluorescence microscopy.^29^ Interestingly, several other anionophores, including **12**, **15** and **19** caused notable decays in fluorescence (Fig. 2a).

To quantify anion transport by different anionophores, we fitted first order exponential functions to the iodide-induced fluorescence decay to measure the initial slope, averaging values over multiple experiments. Fig. 2b and Table 1 summarise the data. Consistent with our previous results,^29^ we observed a wide range of anion transport activity. Some anionophores demonstrated little or no anion transport in YFP-FRT cells (*e.g.***1–3**), others exhibited intermediate levels (*e.g.***13**, **20** and **22**), whereas **19**, **12**, and particularly **15** possessed levels of activity similar to or greater than that of **11** (Fig. 2b). We therefore selected for further study **11**, **12**, **15** and **19**.

### Anion transport by anionophores in CF cells

To develop anionophores as a potential therapy for CF, it is necessary to demonstrate efficacy in epithelial tissues affected by the disease, particularly the respiratory airways, the major site of disease in CF.^7^ To begin to test anionophores in CF airway epithelial cells, we utilised the CF bronchial epithelial cell line CFBE41o^–^ engineered to express YFP-H148Q/I152L (YFP-CFBE cells).^34^,^35^ One advantage of using YFP-CFBE cells is that they endogenously express the Ca^2+^-activated Cl^–^ channel (CaCC), unlike YFP-FRT cells.^33^ Thus, using YFP-CFBE cells, we directly compared anion transport by anionophores with that of the CaCC, an alternative pathway to restore anion transport in CF.^13^

**Fig. 3:**
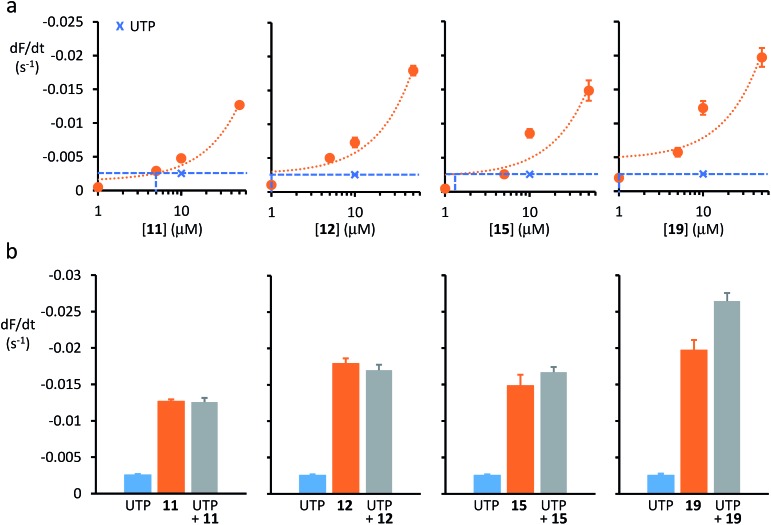
Anion transport by anionophores and CaCC activation in CF cells. (a) Relationship between anionophore concentration and anion transport activity in YFP-CFBE cells for **11**, **12**, **15** and **19**. Crosses and dashed lines indicate the amount of anion transport mediated by CaCC activation with UTP (1 mM). For representative time courses of cell fluorescence from YFP-CFBE cells treated with test anionophores, see ESI Fig. S16.† (b) Anion transport by YFP-CFBE cells treated with anionophores (50 μM) and UTP (1 mM) individually or together. Data are means ± SEM (*n* = 12–64 from at least four independent experiments); in (a), dotted lines are the fit of first-order functions to mean data.

Fig. 3a demonstrates that **11**, **12**, **15** and **19** mediated anion transport in YFP-CFBE cells. For each anionophore, raising the transporter concentration increased the rate of fluorescence decay and hence, anion transport activity. ESI Fig. S16† shows that **11** and **12** exhibited identical concentration–response relationships in YFP-CFBE and YFP-FRT cells, that of **19** was similar in both cell types, but **15** was less efficacious in YFP-CFBE cells. These variations in behaviour may result from the different physical properties of the anionophores. For example, the poorly deliverable **15** may not be able to penetrate the YFP-CFBE cells, given their barrier function in the respiratory airways.

Anion transport by CaCC in YFP-CFBE cells was stimulated using the P2Y (purinergic G protein-coupled) receptor agonist UTP (for chemical structure, see ESI Fig. S15†), which activates the channel by elevating the intracellular Ca^2+^ concentration (Fig. 3).^13^ Comparison of anion transport by anionophores and CaCCs (Fig. 3) reveals two important points. First, low concentrations of anionophores elicited anion transport equivalent to that achieved by CaCC activation. Fig. 3a demonstrates that the anionophore concentrations matching the effect of UTP (1 mM) were **19** (1 μM) = **15** (1 μM) = **12** (1 μM) ≥ **11** (5 μM). Second, for the *trans*-decalin anionophores **11**, **12** and **15** CaCC activation was either without effect or weakly additive to anionophore-mediated anion transport (Fig. 3b). By contrast, for the tren-based compound **19**, CaCC activation was strongly additive to anionophore-mediated anion transport (Fig. 3b). As a control, we tested the effect of the CaCC inhibitor CaCC_inh_-A01 36 (for chemical structure, see ESI Fig. S15†). Addition of CaCC_inh_-A01 nullified the effect of UTP in the case of the *trans*-decalins **11**, **12** and **15** (ESI Fig. S17†). In the case of **19**, CaCC_inh_-A01 also showed evidence of anionophore inhibition (ESI Fig. S17†). This effect is unlikely to reflect a specific interaction, and may suggest that **19** is vulnerable to inhibition by aromatic carboxylic acids. Taken together, the data suggest that anion transport by anionophores in YFP-CFBE cells is independent of CaCC activation and for **19** it is additive to that of CaCC.

### Anion transport by anionophores is additive to that of CFTR

Next, we sought to learn whether the activities of **11**, **12**, **15**, and **19** are additive to that of CFTR. For these studies, we used FRT cells co-expressing wild-type human CFTR and YFP-H148Q/I152L (WT-CFTR-YFP-FRT cells).^28^ ESI Fig. S19A† shows representative time courses of fluorescence decay for the anionophores **11**, **12**, **15**, and **19** (all tested at 50 μM) in the absence and presence of the cAMP agonist forskolin (10 μM) and the CFTR inhibitor CFTR_inh_-172 (10 μM),^37^ while ESI Fig. S19B† summarises data from multiple experiments (for chemical structures of CFTR modulators, see ESI Fig. S18†). Forskolin robustly stimulated anion transport in WT-CFTR-YFP-FRT cells and the response was inhibited, albeit incompletely by CFTR_inh_-172 (ESI Fig. S19†). ESI Fig. S19† demonstrates that anion transport by each of the anionophores was additive to that of forskolin. CFTR_inh_-172 was without effect on anion transport by **11** and **12**, but inhibited the activity of **19** (*P* < 0.001) and caused a small, albeit significant, increase in anion transport by **15** (*P* < 0.001) (ESI Fig. S19†). This latter effect is difficult to explain and merits further investigation.

To learn whether anionophores are additive to small molecules that rescue disease-causing CFTR variants, we used FRT cells co-expressing F508del-CFTR, the predominant CF mutation and YFP-H148Q/I152L (F508del-CFTR-YFP-FRT cells).^28^ To rescue the plasma membrane expression of F508del-CFTR, we used low temperature or the clinically-licensed CFTR corrector lumacaftor,^38^,^39^ while to increase its activity, we used the clinically-licensed CFTR potentiator ivacaftor.^40^

**Fig. 4:**
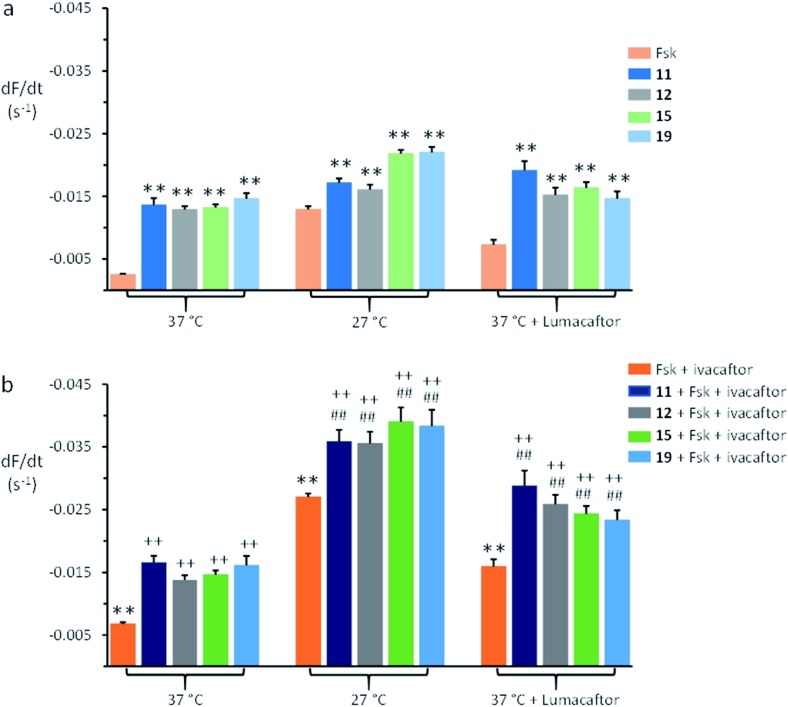
Anion transport by anionophores and CFTR modulators in FRT cells expressing F508del-CFTR. (a) Magnitude of anion transport generated by test anionophores (50 μM) compared to that achieved by stimulating F508del-CFTR with forskolin (Fsk; 10 μM) for the indicated treatments of F508del-CFTR-YFP-FRT cells. (b) Magnitude of anion transport generated by test anionophores (50 μM) together with F508del-CFTR stimulation with forskolin (10 μM) and potentiation with ivacaftor (1 μM) compared to the action of forskolin and ivacaftor on F508del-CFTR for the indicated treatments of F508del-CFTR-YFP-FRT cells. For representative time courses of cell fluorescence, see ESI Fig. S20.† Fluorescence quenching by the anionophore vehicle (DMSO, 0.5% v/v) was subtracted from each test measurement. Data are means ± SEM (*n* = 20–52 from at least four independent experiments); **, *P* < 0.01 *vs.* forskolin; ^##^, *P* < 0.01 *vs.* anionophore; ^++^, *P* < 0.01 *vs.* forskolin + ivacaftor.

Fig. 4 shows summary anion transport data for control F508del-CFTR-YFP-FRT cells grown at 37 °C and cells incubated at 27 °C for 24 h or pre-treated with lumacaftor (3 μM) at 37 °C for 24 h to rescue the plasma membrane expression of F508del-CFTR. Representative time courses of fluorescence decay are shown in ESI Fig. S20.† The anionophores **11**, **12**, **15**, and **19** all mediated anion transport in control F508del-CFTR-YFP-FRT cells grown at 37 °C, whereas forskolin (10 μM) had little effect (Fig. 4a). Low temperature incubation or treatment with lumacaftor enhanced forskolin-mediated Cl^–^ transport by F508del-CFTR, but had little or no effect on anionophore-mediated anion transport (Fig. 4a).

Fig. 4 demonstrates that following rescue of its plasma membrane expression, ivacaftor (1 μM) increased the amount of anion transport achieved by F508del-CFTR expressing FRT cells compared to the action of forskolin (10 μM). When added together with forskolin and ivacaftor, the anionophores **11**, **12**, **15**, and **19** increased further the amount of anion transport generated by F508del-CFTR-YFP-FRT cells (Fig. 4b). Comparison of the magnitude of anion transport in the absence and presence of anionophores demonstrates that their effects were additive to those of forskolin and ivacaftor (Fig. 4b). Of note, similar results were recently reported using F508del-CFTR-YFP-FRT cells treated with lumacaftor, ivacaftor and anionophores derived from prodigiosin and tambjamine.^41^ Thus, anionophores mediate anion transport in cells expressing wild-type and F508del-CFTR and their effects are additive to the action of drugs that rescue F508del-CFTR.

### Anionophore delivery, persistence and efficacy

In previous work,^29^ we demonstrated that the anionophore **11** remains active at the plasma membrane for >2 h. To investigate further the relationship between anionophore delivery to cell membranes, the longevity of their action and their efficacy, we undertook two types of experiment. First, using the microplate reader, we tested the effects of different incubation periods on anion transport by **11**, **12**, and **19** (each tested at 1, 10 and 50 μM). Second, using fluorescence microscopy, we examined the persistence of anion transport by the two most promising anionophores, **11** and **12**, (both tested at 10 μM) after different incubation periods. Because of its reduced deliverability, we did not use **15** in these experiments. Several conclusions can be drawn from the data in ESI Fig. S21 and S22.† First, anion transport by anionophores is concentration-dependent. Second, increasing the incubation period improves anion transport by some, but not all anionophores. Third, for at least two anionophores, **11** and **12**, their effects show noticeable persistence.

### Cytotoxicity of anionophores in CF cells

We previously evaluated the cytotoxicity of **11** and a related decalin bis-urea in three different epithelial cell lines (MDCK, FRT and HeLa), using the reagent XTT to measure mitochondrial activity.^29^ Employing the same method, we assessed the cytotoxicity of **11**, **12**, **15**, and **19** in YFP-CFBE cells. We also examined overall toxicity and the induction of apoptosis in YFP-FRT cells, using the IncuCyte ZOOM™ (Essen Bioscience) live-cell analysis system. The anionophores **15**, **12**, and **19** showed some signs of cytotoxicity in YFP-CFBE cells at the highest concentrations tested with **19** (50 μM) giving the strongest effect (ESI Fig. S23†). The IncuCyte ZOOM™ measurements revealed some toxicity for all four compounds at 50 μM, but the effects were strongly delayed for **11** (ESI Fig. S24†). At 10 μM, **11** showed no toxicity while **12**, **15** and **19** caused some cell death. Notably, the apoptosis assay gave negative results for **11** at all concentrations tested (ESI Fig. S25†), while **12**, **15** and **19** all yielded positive effects. The results support our earlier conclusion that the toxicity of these compounds is not simply related to anion transport ability, and that non-toxic anion transport is a realistic possibility.

## Discussion

This study identified three anionophores, **12**, **15**, and **19**, with promising biological activity, while confirming the potential of previously-studied **11**. The agents were selected from a total of 22 molecules capable of anion transport in synthetic vesicles. Given the general similarity of the molecules tested, in that all bind anions through arrays of H-bond donor groups, the results show striking variations in biological activity. Effectiveness in cells is presumably dependent on two factors: (i) the intrinsic ability of the agent to transport anions, and (ii) the ability of compounds to migrate to the membranes so that they can exert their effects. These can be assessed independently in synthetic vesicles through measurements of specific initial transport rates [*I*], and deliverability *D* (see Table 1). To some extent the performance in LUVs correlates with that in cells. Thus, most of the less active compounds show either low [*I*] or low *D* in LUVs (Table 1). This said, of the four most active compounds, two perform relatively poorly in one or other of the LUV assays; **11** gives [*I*] = 22 s^–1^, which is towards the low end of the scale, while **15** gives *D* = 0.41. The divergence between performance in cells and behaviour in LUVs may be an important topic for future study.

In terms of structure–activity relationships, the *trans*-decalin scaffold continues to be successful, being present in **11**, **12** and **15**. Previous work^19^,^20^,^29^ has highlighted the effectiveness of this system for transport in both vesicles and cells, and also for tunability towards drug-like properties (*e.g.* small molecular weight, controlled lipophilicity). Comparison of results for the six decalin anionophores studied provides insight into the structural features which enhance biological activity. First, the length of the alkyl side chain; where comparisons can be made (*e.g.***12***vs.***13** and **15***vs.***16**), the ethyl esters are far more active than the corresponding octyl esters. This runs counter to the trend observed in vesicle experiments,^19^ and probably reflects the poor deliverability of the longer-chain esters. Indeed, we have previously shown that **16** is far more effective when presented to cells using a coiled-coil-based delivery system.^42^ Second, substitution of ureas by thioureas often improves anion transport in LUVs.^16^,^20^ A possible reason is that urea oxygens are good H-bond acceptors, binding to water molecules and thus are less mobile within membranes.^20^ Accordingly, we find here that **12**, a bis-thiourea decalin, possessed better biological activity than **11**, a bis-urea decalin. Third, fluorination improves anion transport by anionophores (*e.g.***12***vs.***15**, **18***vs.***19** and **21***vs.***22**), probably by enhancing both lipophilicity and anion affinity.^20^,^43^

While the *trans*-decalins feature strongly among the most active agents, other scaffolds can also be effective. Anionophore **19**, one of the four most promising agents, belongs to the tren-based family,^16^,^25^ while anthracenes **20** and **22** 24 are just slightly less effective. Again, it seems that fluorination enhances transport activity; both **19** and **22** contain multiple trifluoromethyl substituents. None of the squaramides **1–3**,^22^ hexa-substituted benzenes **4–7**,^23^ or cyclohexane **8** 21 proved usefully active, probably due to poor deliverability. The squaramides tend to be insoluble, while compounds **4–8** are all highly lipophilic. It seems likely that, in many cases, high lipophilicity causes agents to precipitate in a form which hinders access to cell membranes. This would also account for the failure of octyl-substituted decalins **13** and **16**, as discussed above.

Throughout this study, deliverability emerges as a key factor which can limit the biological activity of anionophores. To investigate anionophore delivery to cells, we varied the period cells were incubated with anionophores **11**, **12** and **19** prior to assaying anion transport. For **12** and **19** prolonged incubation periods enhanced noticeably anion transport, but for **11** there was little improvement. It thus seems that **12** takes longer to reach cell membranes, consistent with its relatively low deliverability in vesicles. Like **11** (ref. 29; present results), **12** demonstrated noticeable persistence, mediating anion transport for up to 2 h after anionophore delivery to FRT cells, despite the short incubation period and continuous perfusion of cells with physiological salt solutions. Although it is known that decalin anionophores do not leach from LUVs,^19^ the fate of anionophores delivered to cells remains to be determined.

Cytotoxicity studies of anionophores have produced conflicting results. Calixpyrroles and certain squaramides transport anions in LUVs, but treatment of cells with these agents leads to apoptotic cell death.^44^–^46^ By contrast, the decalin **11** has potent biological activity yet is almost without toxic effects on four different epithelial cell lines (ref. 29; present results). Interestingly, the tren **19** was cytotoxic in some, but not all cancer cells tested and was without toxic effects on CF cells at concentrations that mediated anion transport greater than that achieved by CaCC activation (ref. 16; present results). Nevertheless, higher concentrations of **19** as well as **12** and **15** exhibited some cytotoxicity on CF cells and initiated apoptosis in FRT cells. Taken together, these and other data^44^–^46^ argue that the cytotoxicity of anionophores is compound specific and anion transport, by itself, does not trigger cell death. They also support a variety of potential therapeutic applications for anionophores, some as anticancer and antimicrobial agents,^45^–^47^ but others as CF therapeutics.^29^,^41^,^48^

If anionophores are to be used as research tools, it is important to establish their sensitivity to small molecules that inhibit endogenous anion transport systems. We therefore studied the effects of CaCC_inh_-A01 (active against CaCC) and CFTR_inh_-172 (active against CFTR),^36^,^37^ on transport by **11**, **12**, **15** and **19**. **11** and **12** were unaffected by either CaCC_inh_-A01 or CFTR_inh_-172, further demonstrating that their activity is independent of endogenous pathways for anion transport in cells. However, **19** was inhibited by both CaCC_inh_-A01 and CFTR_inh_-172, possibly because the tren scaffold is more flexible and accessible to small molecule inhibitors.^49^ Surprisingly, CFTR_inh_-172 increased anion transport by **15** by a small but significant degree. Further studies are required to understand this effect.

In the present study, UTP activated little CaCC activity in CF cells, probably due to low levels of expression of the CaCC TMEM16A.^34^,^35^ By contrast, in the pro-inflammatory conditions of the CF lung, CaCC activity is enhanced strongly by increased TMEM16A expression.^35^,^50^ Encouragingly, the present results show that the activity of some anionophores, particularly the tren **19**, is additive to CaCC activation. These data argue that some anionophores might be combined with small molecules that directly target the CaCC TMEM16A in airway epithelia to achieve sustained CaCC activity mimicking CFTR function.^13^ Future studies should explore this innovative approach to CFTR bypass therapy.

Since 2012, drugs that target disease-causing variants in CFTR have become available to CF patients.^9^–^11^ For individuals with the F508del defect and those with some rare variants, combination therapy with two or more CFTR correctors (drugs that deliver variants to the cell membrane), together with a potentiator (drugs that boost channel activity), may prove at least partially effective.^51^–^53^ However, not all individuals living with CF are likely to benefit from combination therapy with CFTR modulators. These individuals will require CFTR variant-independent therapies to restore anion transport to affected epithelial tissues. Moreover, patients who are helped by treatment with CFTR correctors/potentiators may benefit further from complementary approaches, such as anion-transporting small molecules, which restore bacterial killing to CF cells.^54^ Encouragingly, the present results and ref. 41 demonstrate that the action of anionophores is additive to the clinically-licensed CFTR modulators lumacaftor and ivacaftor, raising the possibility that combination therapy with anionophores and CFTR modulators might be used to treat CF patients. While ivacaftor and lumacaftor are orally bioavailable drugs,^39^,^40^ we envisage that anionophores would be delivered to the lungs of CF patients by aerosolization as for inhaled antibiotics and gene therapy vectors.^7^,^55^

## Conclusions

In conclusion, we show that anionophore evaluation using fluorescence emission from halide-sensitive YFP can be adapted for use with a microplate reader to accelerate the identification of anionophores with promising biological activity. We exploit the method to test anionophores in several cell lines including CF airway epithelial cells, a key target for CF therapeutics. We demonstrate that our most promising anionophore **11** is effective in YFP-modified CF airway epithelial cells, where it is without cytotoxic effects. Of note, the activity of some anionophores is additive to CaCC activation, while the activity of **11** and three other biologically-active anionophores is additive to rescue of the predominant CF-causing variant F508del-CFTR using the clinically-licensed CFTR modulators lumacaftor and ivacaftor. These data encourage us to believe that anionophores might be developed to provide therapeutics for all individuals with CF. They might be used alone or as part of a combination therapy with either small molecule CaCC potentiators or CFTR modulators.

## Experimental

### Cells and cell culture

FRT cells stably expressing YFP-H148Q/I152L^28^ were generous gifts of AS Verkman (University of California, San Francisco), while CFBE41o^–^ cells^34^ stably expressing YFP were a generous gift of LJV Galietta (Telethon Institute for Genetics and Medicine). These cells were cultured as described in the ESI.†

### Reagents

New anionophores (**9**, **10**, **12** and **13**) were synthesized as described in the ESI;† all other anionophores and chemicals were prepared and used as specified in the references (Table 1) and ESI.†

### Fluorescence plate reader assay

Anionophore-mediated anion transport was quantified by measuring I^–^-induced quenching of YFP fluorescence at 37 °C using a microplate reader following the method of Galietta *et al.*^27^ as described in the ESI.†

### Statistics

Results are expressed as means ± SEM of *n* observations. To compare sets of data, we used Student's *t*-test. Differences were considered statistically significant when *P* < 0.05. All statistical tests were performed using SigmaPlot 12 (Systat Software Inc., San Jose, CA, USA).

## Conflicts of interest

There are no conflicts to declare.

## Supplementary Material

Supplementary information

Infographic
