# Supplementary material for: Anion carriers as potential treatments for cystic fibrosis: transport in cystic fibrosis cells, and additivity to channel-targeting drugs
Source: Chem Sci. 2019 Oct 2;10(42):9663–72. doi: 10.1039/c9sc04242c (PMC6984391; doi:10.1039/c9sc04242c)
Supplement: Supplementary file 1 [file SC-010-C9SC04242C-s001.pdf]

Electronic Supplementary Material (ESI) for Chemical Science.
This journal is © The Royal Society of Chemistry 2019

Electronic Supplementary Information

Anion carriers as potential treatments for cystic fibrosis: transport in cystic fibrosis cells, and additivity to channel-targeting drugs

Hongyu Li[1], Hennie Valkenier[2 †], Abigail G. Thorne[2], Christopher M. Dias[2], James A. Cooper[2], Marion Kieffer[2], Nathalie Busschaert[3 ‡], Philip A. Gale[3 § *], David N. Sheppard[1 *] and Anthony P. Davis[2 *]

[1]School of Physiology, Pharmacology and Neuroscience, University of Bristol, Biomedical Sciences Building, University Walk, Bristol BS8 1TD, UK,
[2]School of Chemistry, University of Bristol, Cantock's Close, Bristol BS8 1TS, UK
and
[3]Chemistry, University of Southampton, Southampton SO17 1BJ, UK

[†]Present address: Engineering of Molecular NanoSystems, Université libre de Bruxelles, 50 avenue F.D. Roosevelt, B-1050 Brussels, Belgium
[‡]Present address: Department of Chemistry, Tulane University, New Orleans, LA 70118, USA
[§]Present address: The University of Sydney, School of Chemistry (F11), NSW 2006, Australia

*Address Correspondence to: A.P. Davis, Ph.D.
E-mail: Anthony.Davis@bristol.ac.uk)

D.N. Sheppard, Ph.D.
E-mail: D.N.Sheppard@bristol.ac.uk
and
P.A. Gale, D.Phil., D.Sc.
E-mail: philip.gale@sydney.edu.au

Contents

1. Synthesis ... 3

General Synthetic Experimental Information. ... 3

Synthesis of anionophores **9** and **10** ... 4

Synthesis of anionophore **12** ... 12

Synthesis of anionophore **13** ... 13

2. Binding studies ... 15

Measurement of affinities to $Et_4N^+Cl^-$ in chloroform through extraction from water ... 15

Measurement of affinity of **12** to $Bu_4N^+Cl^-$ in DMSO-d_6/H_2O (200:1) ... 17

3. Transport studies in vesicles ... 19

Transport measurements in vesicles with preincorporated anionophores. ... 19

Deliverability of anionophores to vesicles. ... 20

4. Biological Data ... 26

Materials and Methods ... 26

Additivity of anion transport by anionophores and epithelial Cl^- channels ... 29

Anionophore delivery, persistence and efficacy ... 35

Toxicity studies ... 38

5. References ... 42

1. Synthesis

The new decalin-based anionophores reported here (**9**, **10**, **12**, and **13**) were synthesised upon deprotection of scaffolds **A**-**C**, followed by the reaction with the appropriate iso(thio)cyanate, using the methodology we reported previously.[1,2]

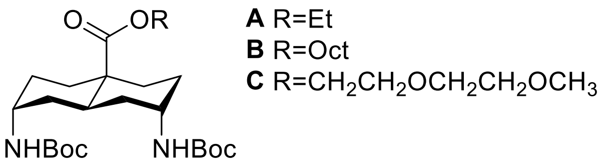

The synthesis of ethyl-ester scaffold **A** and octyl-ester scaffold **B** were described in references 1 and 2, respectively. The 2-(2-methoxy)ethoxy ethane-ester scaffold **C** was synthesised using a similar strategy as used for octyl-ester scaffold **B** and is detailed below.

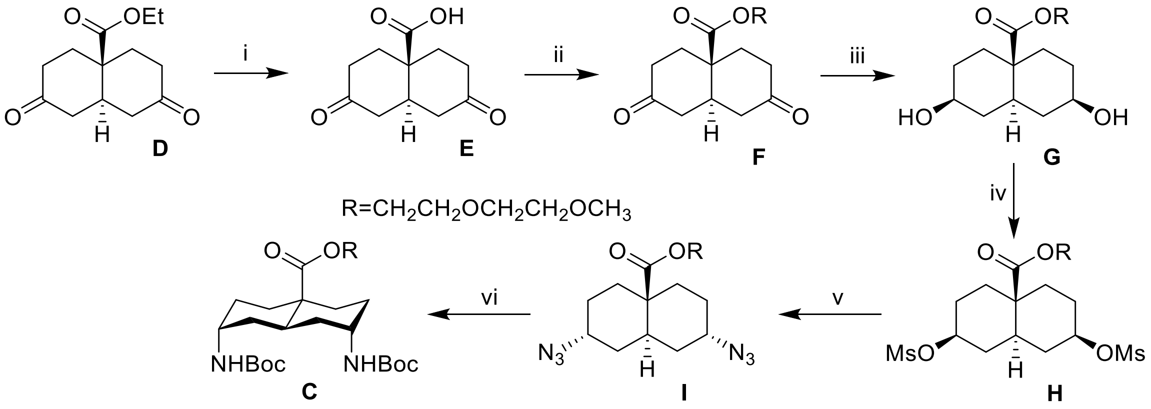

Scheme S1. Reagents and conditions for the synthesis of *trans*-decalin scaffold **C**: (i) HCl, H_2O, reflux, 95%. (ii) bromo-2-(2-methoxy)ethoxy ethane, K_2CO_3, DMF, RT, 28%. (iii) $NaBH_4$, MeOH, 0 °C. (iv) MsCl, DIPEA, MeOH, 0 °C –RT, 51% over 2 steps. (v) NaN_3, DMF, 90 °C. (vi) a. PMe_3, THF, b. Boc-ON, THF, 50 °C, 26% over two steps.

General Synthetic Experimental Information.

All commercially available compounds were used without further purification. THF and dichloromethane were dried by passing through a modified Grubbs system[3] with an alumina column. Flash column chromatography was performed using silica gel (Aldrich, pore size 60 Å, particle size 40-63 µm) as the absorbent. Routine monitoring of reactions was performed using precoated silica gel TLC plates (Merck silica gel 60 F_{254}). Spots were visualised by UV light or stained with an ethanolic solution of phosphomolybdic acid. ^1H and ^{13}C NMR spectra were recorded using ECS 400, Varian 400, Varian 500a (carbon sensitive), or Varian 500b (proton sensitive) spectrometers. All chemical shifts (δ) are quoted in parts per million (ppm)

and the residual solvent signal was used to reference the spectrum. Signals are described as singlet (s), broad singlet (bs), doublet (d), triplet (t), triple doublet (td), quartet (q), and multiplet peaks (m). Mass spectra by electrospray ionisation (positive mode) were recorded on a Bruker MicrOTOF. Infrared spectra were recorded on a Perkin Elmer Spectrum Two FT-IR.

Synthesis of anionophores **9** and **10**

Compound **E**

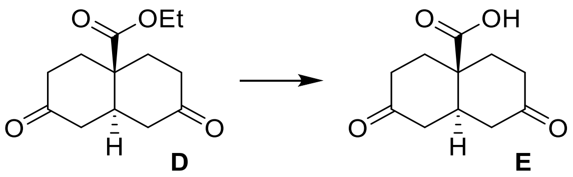

The diketone ethyl ester **D** (4.8 g, 20.14 mmol) was stirred in 4 M HCl (300 mL) and refluxed at 100 °C for 24 hours. The reaction mixture was evacuated to dryness to yield a dark brown oil, which was dissolved in DCM (200 mL) and washed with water (2 x 50 mL). The aqueous phase was washed with ethyl acetate (50 mL) and the organic phases were combined and dried over $MgSO_4$. The organic phase was evacuated to dryness giving a light brown solid **E** (4.04 g, 19.22 mmol, 95%); ν_{max} (neat)/ cm^{-1}: 3415 (w, OH), 2948 (w, CH), 2882 (w, CH), 1713 (s, C=O), 1370 (w), 11230 (w), 1187 (w); MS(ESI): *m/z* calculated for $C_{11}H_{13}O_4$ $[M - H]^-$: 209.2, found 209.1.

Compound **F**

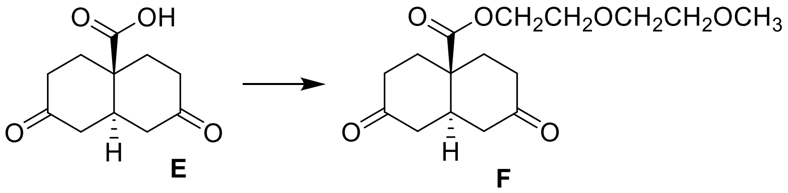

The crude acid **E** (1.5 g, 7.13 mmol) was dissolved in dry DMF (10 mL) and anhydrous potassium carbonate was added (3.45 g, 24.96 mmol, 3.5 equiv.). The mixture was stirred and bromo-2-(2-methoxy)ethoxy ethane (1.9 mL, 14.12 mmol, 2.00 equiv.) was added via a syringe at ambient temperature. The reaction mixture was stirred at ambient temperature for 17 hours and the potassium carbonate was removed by gravity filtration. The solvent was removed under vacuum and the crude oil was dissolved in ethyl acetate (40 mL) and washed with saturated $NaHCO_3$ (2 x 25 mL) and with water (25 mL). The aqueous phase was extracted with ethyl

acetate (2 x 125 mL) and diethyl ether (2 x 125 mL), and all combined organic phase were dried with $MgSO_4$, and the solvent was removed under pressure to afford an oil. The crude oil was purified by flash column chromatography (5% methanol/ ethyl acetate) to yield the diketone **F** (616 mg, 1.97 mmol, 28%) as a yellow oil. R_f = 0.54 (5% methanol/ ethyl acetate); ^{1}H NMR (400 MHz, $CDCl_3$): δ = 1.56 – 1.72 (m, 3H), 2.07 – 2.18 (m, 1H), 2.24 (ddd, *J* = 15.4, 4.5, 1.4 Hz, 2H), 2.38 – 2.44 (m, 3H), 2.46 – 2.54 (m, 2H), 2.97 (dd, *J* = 15.4, 13.4 Hz, 2H), 3.34 (d, *J* = 0.4 Hz, 3H), 3.45 – 3.52 (m, 2H), 3.57 – 3.64 (m, 2H), 3.71 – 3.77 (m, 2H), 4.39 – 4.47 (m, 2H); ^{13}C NMR (100 MHz, $CDCl_3$): δ = 35.59, 38.49, 43.54, 44.37, 46.25, 59.02, 63.49, 68.94, 70.33, 71.87, 173.51 and 208.71; ν_{max} (neat)/ cm^{-1}: 2958 (w, CH), 2918 (w, CH), 2867 (w, CH) 1709 (s, C=O), 1193 (s), 1106 (s); MS(ESI): *m/z* calculated for $C_{16}H_{24}O_6H$ [M + H]$^+$: 313.165, found 313.165.

Compound **G**

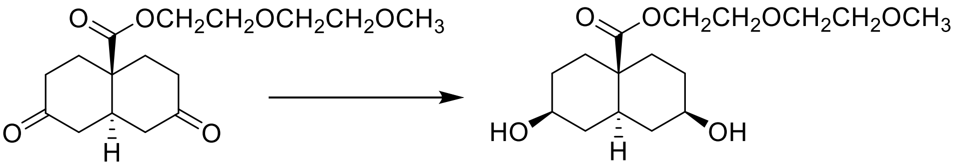

F **G**

Diketone **F** (0.5 g, 1.6 mmol) was dissolved in dry methanol (5 mL) and cooled to 0 °C. Sodium borohydride (127 mg, 3.36 mmol, 2.1 equiv.) was added in one portion and stirred for 2.5 hours. The reaction was quenched with aqueous acetic acid (10%, 6 mL) at 0 °C and stirred for 15 mins. The solvent was removed under reduced pressure and co-evaporated with water (1 x 15 mL) and toluene (3 x 10 mL) to yield crude PEG dialcohol **G**.

Compound **H**

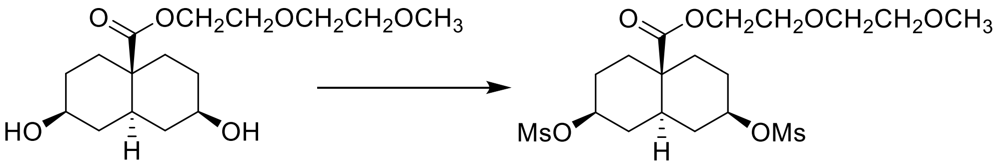

G **H**

The crude oil of **G** (approx. 1.6 mmol) was dissolved in dry THF (10 mL) and the reaction mixture was cooled to 0 °C. DIPEA (1.26 mL, 7.2 mmol, 4.5 equiv.) was added followed by methanesulfonyl chloride (1.24 mL, 16.0 mmol, 10 equiv.) drop wise over 15 mins. The reaction was stirred at 0 °C for 10 mins and then at ambient temperature for 2 hours. The reaction was quenched with saturated $NaHCO_3$ (10 mL) and poured into ethyl acetate (70 mL).

The reaction mixture was washed with saturated $NaHCO_3$ (3 x 10 mL) and brine (10 mL). The aqueous phase was washed with ethyl acetate (50 mL). The combined organic phases were dried over $MgSO_4$ and evacuated to dryness. The crude oil was run over a short silica column (10% MeOH/ DCM) and the eluent was evacuated to dryness. The crude oil was purified by flash chromatography (5% MeOH/ DCM, 70% ethyl acetate/ DCM) to yield the dimesylate **H** (382 mg, 0.81 mmol, 51%, over two steps) as a yellow oil. R_f = 0.60 (2% methanol/ DCM); ^{1}H NMR (400 MHz, $CDCl_3$): δ = 1.16 – 1.27 (m, 2H), 1.34 – 1.46 (m, 1H), 1.47 – 1.60 (m, 3H), 1.92 (ddd, J = 12.2, 6.5, 3.2 Hz, 2H), 2.05 – 2.12 (m, 2H), 2.20 – 2.29 (m, 3H), 2.99 (d, J = 0.4 Hz, 6H), 3.37 (s, 3H), 3.51 – 3.55 (m, 2H), 3.60 – 3.63 (m, 2H), 3.67 – 3.72 (m, 2H), 4.25 – 4.36 (m, 2H) 4.60 – 4.68 (m, 2H); ^{13}C NMR (125 MHz, $CDCl_3$): δ = 29.75, 34.79, 34.91, 38.84, 40.79, 45.99, 59.02, 63.21, 69.00, 70.32, 71.85, 79.82, 173.51; ν_{max} (neat)/ cm^{-1}: 3014 (w, CH), 2938 (w, CH), 2878 (w, CH), 1719 (s, C=O), 1338 (s), 1169 (s), 934 (s); MS(ESI): m/z calculated for $C_{18}H_{32}O_{10}S_2Na$ [M + Na]$^+$: 495.133, found 495.131.

Compound **I**

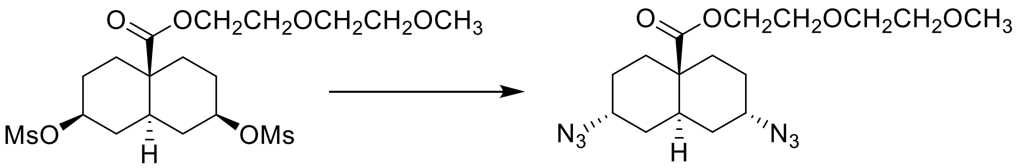

H **I**

Compound **H** (365 mg, 0.77 mmol) and sodium azide (0.92 g, 14.29 mmol, 18.5 equiv.) were stirred in dry DMF (10 mL) and all glass joints were covered with PTFE tape to minimize explosion risk. The reaction mixture was stirred at 90 °C for 22 hours and cooled to ambient temperature, the remaining sodium azide was removed by gravity filtration. The filtrate was evaporated to 6 mL (retaining 5 mL of solvent to minimise explosion risk) and the remaining solution was added to ethyl acetate (20 mL) and washed with water (4 x 10 mL) and the organic phase was dried over $MgSO_4$. The organic phase was evacuated to 6 mL and purified by flash chromatography (66% ethyl acetate/ DCM) to give the diazide **I**. R_f = 0.59 (66% ethyl acetate/ DCM). MS(ESI): m/z calculated for $C_{16}H_{26}N_6O_4Na$ [M + Na]$^+$: 389.191, found 389.191.

2-(2-methoxy)ethoxy ethane-ester scaffold **C**

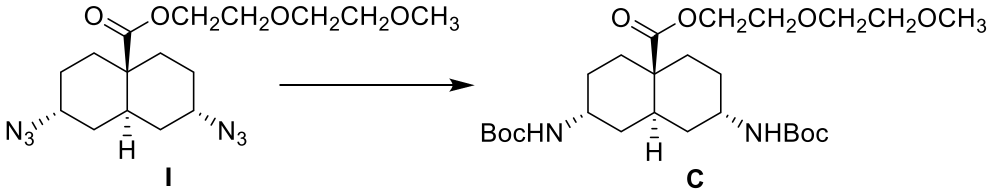

The diazide **I** was transferred as a solution (0.77 mmol) and dry THF (5 mL) and trimethylphosphine (1.0 M solution in THF, 4.62 mL, 4.62 mmol, 6 equiv.) were added drop wise. The reaction mixture was stirred for 2 hours at ambient temperature and cooled to -15 ˚C using an ice salt bath. Boc-ON (0.49 g, 1.99 mmol, 2.58 equiv.) was added portion wise and the mixture was stirred at 0 ˚C for 15 mins after which the ice bath was removed and it was stirred at room temperature for 2 hours and then heated at 50 ˚C for 23 hours. The reaction mixture was cooled to room temperature and additional Boc-ON was added (0.38 g, 1.54 mmol, 2 equiv.) and the mixture was heated at 55 ˚C for 19.5 hours. The reaction mixture was cooled to ambient temperature, poured into water (25 mL) and dissolved in diethyl ether (100 mL). The organic phase was washed with water (2 x 25 mL), dried over $MgSO_4$ and evacuated to dryness. The yellow residue was run over a short column of activated neutral aluminium oxide (1% MeOH/ DCM) and the eluent was evacuated to dryness. The oil was further purified using flash chromatography (1% MeOH/ 40% ethyl acetate/ petroleum spirits (40-60)) to give Boc protected scaffold **C** (103 mg, 0.20 mmol, 26%, over two steps) as an oil that crystallised overnight to form a yellow waxy solid. R_f = 0.45 (1% MeOH/ 40% ethyl acetate/ petroleum spirits (40-60)); ^{1}H NMR (400 MHz, $CDCl_3$): δ = 1.21 – 1.40 (m, 5H), 1.44 (s, 20H), 1.71-1.74 (m, 2H), 1.90-1.94 (m, 2H), 2.13 (td, *J* = 13.6, 4.2 Hz, 2H), 3.35 (s, 3H), 3.47 – 3.52 (m, 2H), 3.56 – 3.61 (m, 2H), 3.64 – 3.69 (m, 2H), 3.80 – 3.93 (m, 2H), 4.23 – 4.28 (m, 2H), 4.82 (d, *J* = 7.1 Hz, 2H); ^{13}C NMR (125 MHz, $CDCl_3$): δ = 27.49, 28.54, 32.26, 33.27, 33.45, 45.93, 47.99, 59.04, 62.86, 69.16, 70.34, 71.90, 79.33, 155.30, 174.40; v_{max} (neat)/ cm^{-1}: 3355 (w, NH), 2969 (w, CH), 2928 (w, CH), 2859 (w, CH), 1711 (s, C=O), 1519 (s), 1365 (s), 1165 (s); MS(ESI): *m/z* calculated for $C_{26}H_{46}N_2O_8Na$ $[M + Na]^+$: 537.315, found 537.31.

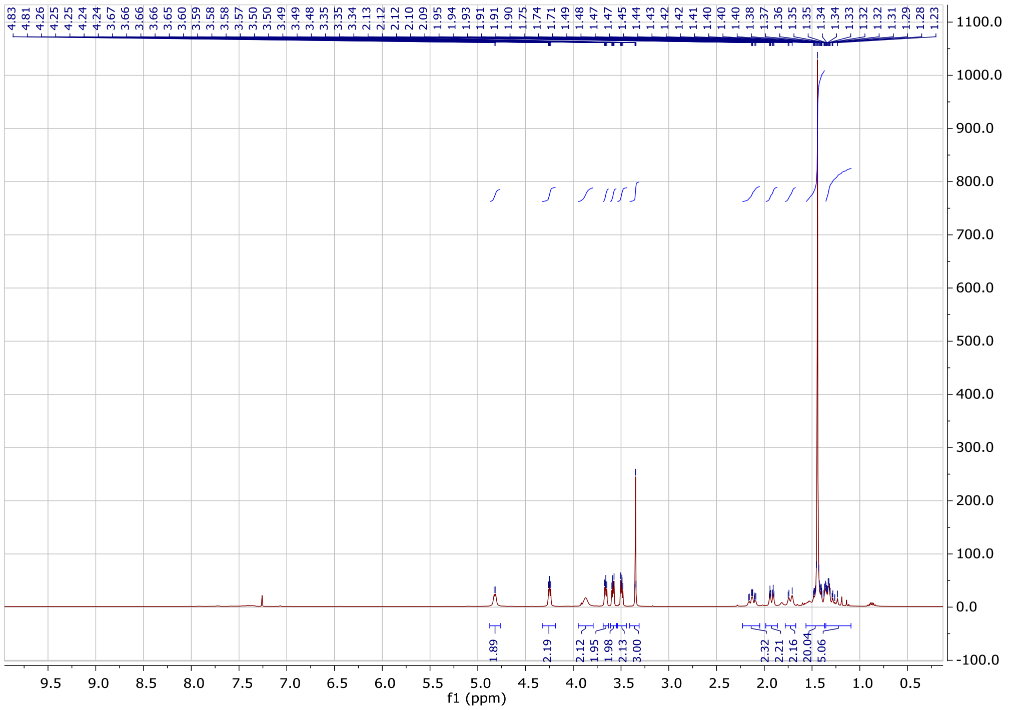

Figure S1. ^{1}H NMR spectrum of 2-(2-methoxy)ethoxy ethane-ester scaffold **C** in acetone-d6.

Anionophore **9**

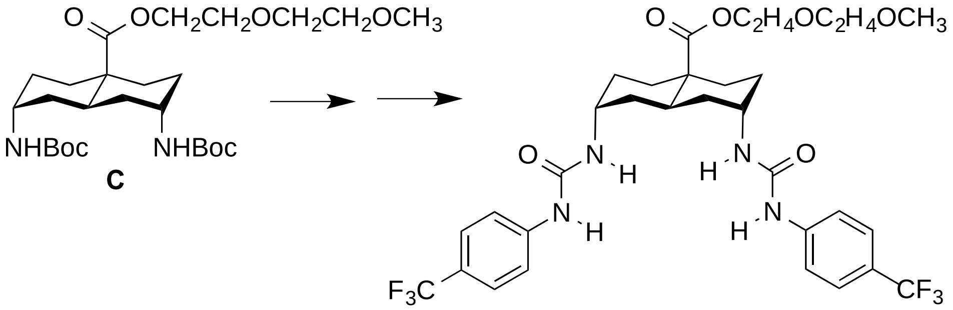

2-(2-methoxy)ethoxy ethane-ester scaffold **C** (50 mg, 0.097 mmol) was dissolved in dry DCM (2.5 mL) and stirred at 0 °C and trifluoroacetic acid (0.75 mL, 9.72 mmol, 100 equiv.) was added drop wise. The reaction mixture was stirred at 0 °C for an hour and then at room temperature for 17 hours. The solvent was removed under vacuum and the residual trifluoroacetic acid was removed by co-evaporation with toluene/MeOH and a yellow oil was isolated and dried at the high vacuum line for 2 hours. The oil was dissolved in dry THF (5 mL) and in turn DMAP (38 mg, 0.32 mmol, 3.25 equiv.), DIPEA (0.5 mL, 2.81 mmol, 29 equiv.) and 4-(trifluoromethyl) phenyl isocyanate (30.5 µl, 0.21 mmol, 2.2 equiv.) were added at room temperature. The reaction mixture was stirred for 17 hour, evacuated to dryness and dissolved in ethyl acetate (50 mL) and subsequently washed with sulphuric acid (0.25 M, 8 mL) and saturated $NaHCO_3$ (8 mL). The organic phase was dried over $MgSO_4$ and evacuated to

dryness on the rotary evaporator. The material was purified by flash column chromatography (5% methanol/ DCM) to give **9** (49 mg, 0.071 mmol, 73%) as a light yellow solid. R_f = 0.47 (5% methanol/ DCM); ^{1}H NMR (500 MHz, acetone-*d*$_6$): δ = 1.46 – 1.65 (m, 6H), 1.78 – 2.00 (m, 5H), 2.23 (td, *J* = 13.6, 4.2 Hz, 2H), 3.32 (s, 3H), 3.47 – 3.54 (m, 2H), 3.59 – 3.65 (m, 2H), 3.68 – 3.73 (m, 2H), 4.05 (dt, *J* = 6.2, 3.0 Hz, 2H), 4.27 – 4.34 (m, 2H), 6.17 (d, *J* = 6.0 Hz, 2H), 7.51 – 7.58 (d, *J* = 8.4 Hz 4H), 7.68 (d, *J* = 8.5 Hz, 4H), 8.15 (s, 2H); ^{13}C NMR (125 MHz, acetone-*d*$_6$): δ = 27.46, 32.28, 33.39, 33.41, 45.61, 48.02, 57.97, 62.80, 68.84, 70.03, 71.80, 117.29, 122.35 (q, *J* = 30.79 Hz), 123.71 (q, *J* = 269.5 Hz), 125.87 (q, *J* = 3.95 Hz), 144.28, 154.34, 174.17; ^{19}F NMR (500 MHz, acetone-*d*$_6$): δ = -62.10; ν_{max} (neat)/ cm^{-1}: 3348 (w, NH), 3064 (w, CH), 2924 (w, CH), 2857 (w, CH), 1663 (s, C=O), 1542 (s), 1319 (s), 1106 (s); MS(ESI): *m/z* calculated for $C_{32}H_{39}F_6N_4O_6$ $[M + H]^+$: 689.27683, found 689.27777.

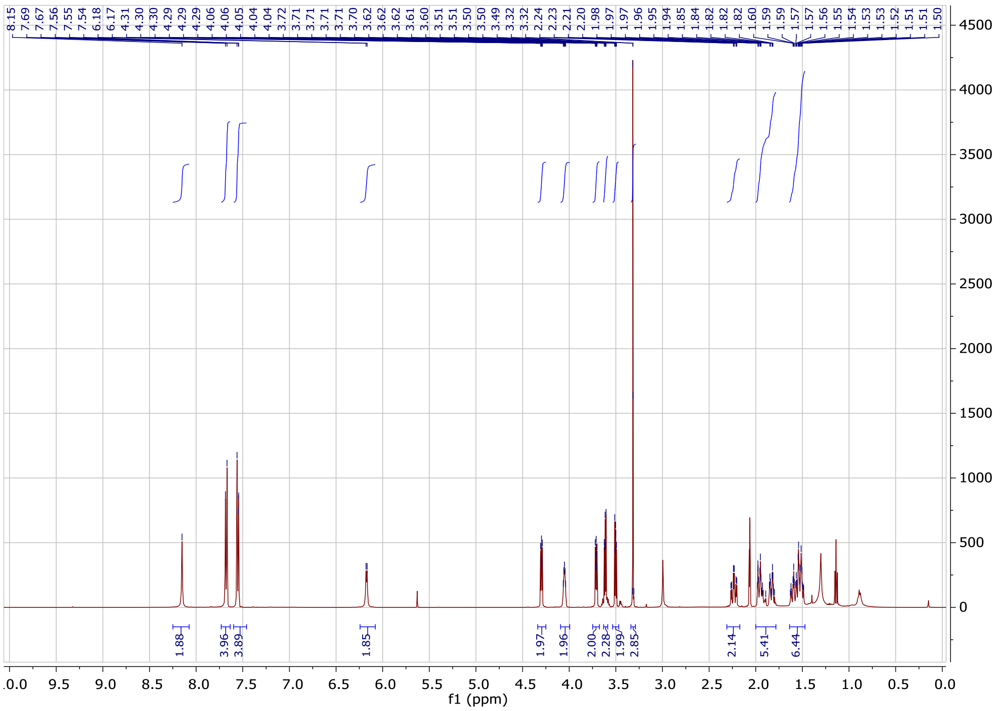

Figure S2. ^{1}H NMR spectrum of **9** in acetone-d$_6$.

Anionophore **10**

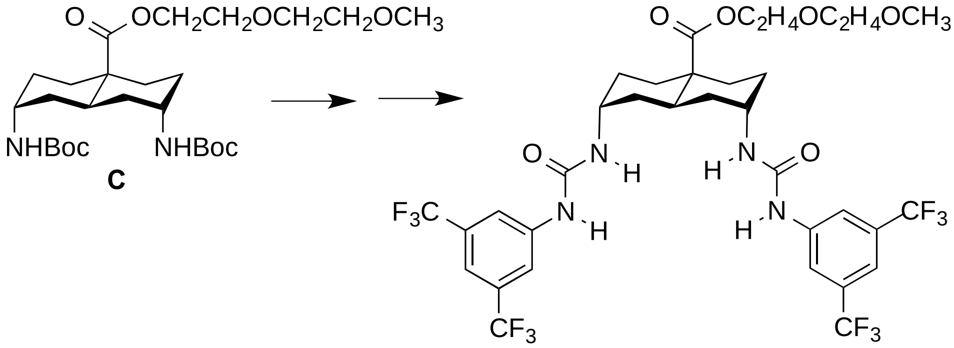

2-(2-methoxy)ethoxy ethane-ester scaffold **C** (50 mg, 0.097 mmol) was dissolved in dry DCM (2.5 mL) and cooled to 0 °C where trifluoroacetic acid (0.75 mL, 9.72 mmol, 100 equiv.) was added drop wise and the reaction mixture was stirred at 0 °C for 1 hour and then at room temperature for 17 hours. The reaction mixture was evacuated to dryness and the trifluoroacetic acid was removed by co-evaporation with toluene (5 x 2 mL). The resulting material was dried at the high vacuum line for 1 hour to afford a light brown oil. The oil was dissolved in dry THF (2 mL) and sequentially DMAP (38 mg, 0.32 mmol, 3.25 equiv.), DIPEA (0.5 mL, 2.81 mmol, 29 equiv.) and 3,5-bis(trifluoromethyl)phenyl isocyanate (36.9 µl, 0.21 mmol, 2.2 equiv.). The reaction mixture was stirred at ambient temperature for 16 hours and then evacuated to dryness and dissolved in ethyl acetate (50 mL). The organic phase was washed with 0.25 M sulphuric acid (8 mL) and saturated $NaHCO_3$ (8 mL), dried over $MgSO_4$ and purified by flash chromatography (6% MeOH/DCM) to afford **10** (53 mg, 0.064 mmol, 66%) as a yellow solid. R_f = 0.32 (10% MeOH/ DCM); ^{1}H NMR (400 MHz, acetone-d_6): δ = 1.42 – 1.70 (m, 6H), 1.79 – 2.02 (m, 5H), 2.23 (td, *J* = 13.6, 4.2 Hz, 2H), 3.31 (s, 3H), 3.39 – 3.55 (m, 2H), 3.55 – 3.65 (m, 2H), 3.64 – 3.75 (m, 2H), 4.00 – 4.06 (m, 2H), 4.18 – 4.34 (m, 2H), 6.21 (d, *J* = 5.8 Hz, 2H), 7.52 (dd, *J* = 2.1, 1.2 Hz, 2H), 7.90 – 8.24 (m, 4H), 8.39 (s, 2H); ^{13}C NMR (125 MHz, acetone-d_6): δ = 27.39, 32.25, 33.26, 33.65, 45.83, 47.97, 57.96, 62.82, 68.84, 70.02, 71.80, 113.86 (m), 117.34 (m), 122.50, (q, *J* = 278.67 Hz), 131.39, (q, *J* = 35.08 Hz) 142.61, 154.33, 174.12; ^{19}F NMR (500 MHz, acetone-d_6): δ = -64.12; ν_{max} (neat)/ cm^{-1}: 3352 (w, NH), 3110 (w, CH), 2928 (w, CH), 2863 (w, CH), 1726 (w, C=O), 1668 (w, C=O), 1553 (w), 1274 (s), 1124 (s); MS(ESI): *m/z* calculated for $C_{34}H_{36}F_{12}N_4O_6Na$ $[M + Na]^+$: 847.2341, found 847.2388.

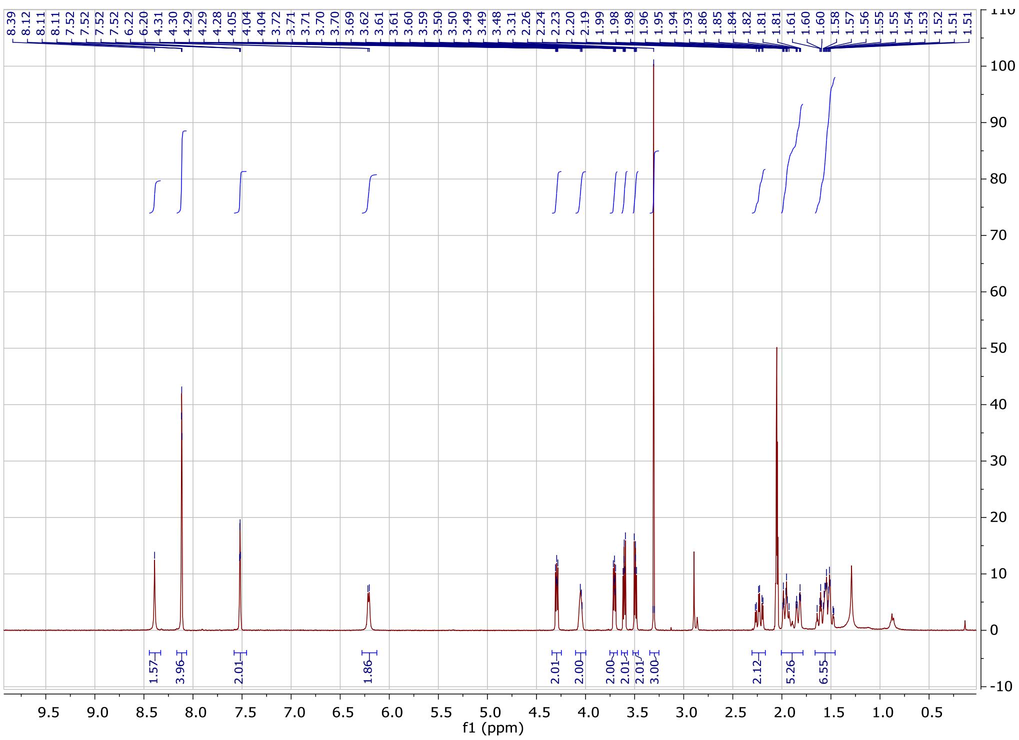

Figure S3. ^{1}H NMR spectrum of **10** in acetone-d_6.

Synthesis of anionophore **12**

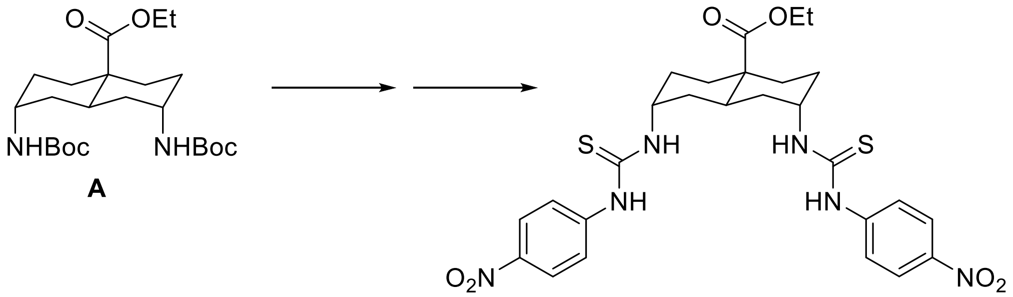

Ethyl-ester scaffold **A** (73.2 mg, 166 µmol) was placed in a 25 mL round bottom flask under nitrogen and dry dichloromethane (6 mL) was added. The resulting solution was cooled with an ice bath and trifluoroacetic acid (1.5 mL, 20 mmol, 122 eqv.) was added drop wise. The reaction mixture was stirred at 0 ˚C for 30 min. and at room temperature for 15 hours. The solvent and trifluoroacetic acid were removed by coevaporation with toluene and methanol. The residue was combined with DMAP (53.6 mg, 478 µmol, 2.9 eqv.), 4-nitrophenyl isothiocyanate (66.0 mg, 336 µmol, 2.2 eqv.), dry THF (15 mL), and DIPEA (1.5 mL, 9.1 mmol, 55 eqv.) and the reaction mixture was stirred for 21 hours at room temperature. The solvent was evaporated and the crude material was redissolved in ethyl acetate (150 mL), washed with aqueous sulphuric acid (0.25 M, 50 mL) and saturated $NaHCO_3$ (50 mL), dried over $MgSO_4$, filtered and concentrated to dryness. The resulting material was purified by column chromatography over silica gel eluted with 3-4% methanol in dichloromethane, yielding 53.4 mg (89 µmol, 54%) of transporter **12** as a yellow solid. R_f = 0.19 (3% MeOH in DCM). ^{1}H NMR (400 MHz, acetone-d_6) δ = 9.25 (bs, 2H), 8.17 (d, *J* = 9.2 Hz, 4H), 7.93 (d, *J* = 9.2 Hz, 4H), 7.72 (bs, 2H), 4.59 (bs, 2H), 4.21 (q, *J* = 7.1 Hz, 2H), 2.25 (td, *J* = 13.7, 4.3 Hz, 2H), 2.04 – 1.85 (m, 5H), 1.81 – 1.70 (m, 2H), 1.65 – 1.49 (m, 4H), 1.28 (t, *J* = 7.1 Hz, 3H). ^{13}C NMR (126 MHz, acetone-d_6) δ = 180.86, 174.78, 147.07, 143.61, 125.17, 121.39, 60.74, 50.53, 48.35, 35.60, 33.48, 33.25, 27.54, 14.56. ν_{max} (neat)/ cm^{-1}: 3376, 3304, 2933, 1716, 1702, 1597, 1496, 1324, 1199, 1111, 1032, 849, 748. HRMS (ESI): m/z calculated for $C_{27}H_{32}N_6NaO_6S_2^+$ [M + Na]$^+$: 623.1717, found 623.1702.

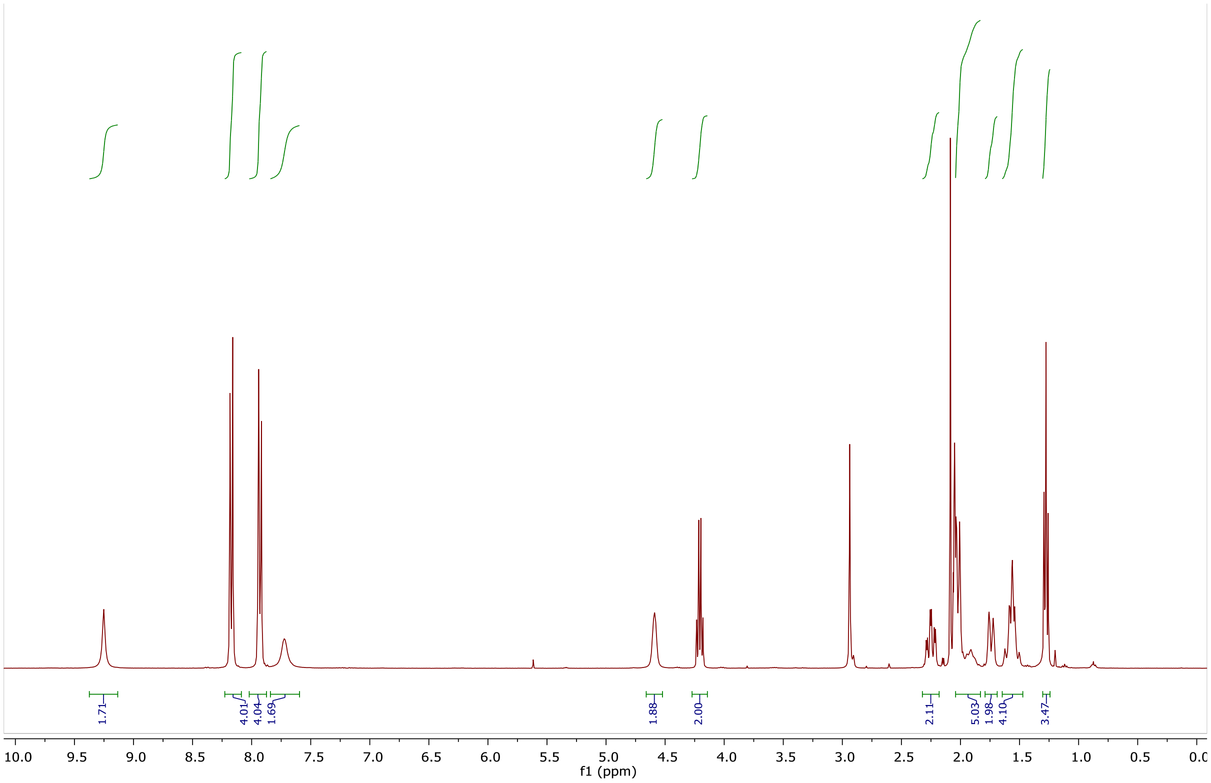

Figure S4. ^{1}H NMR spectrum of **12** in acetone-d_6.

Synthesis of anionophore **13**

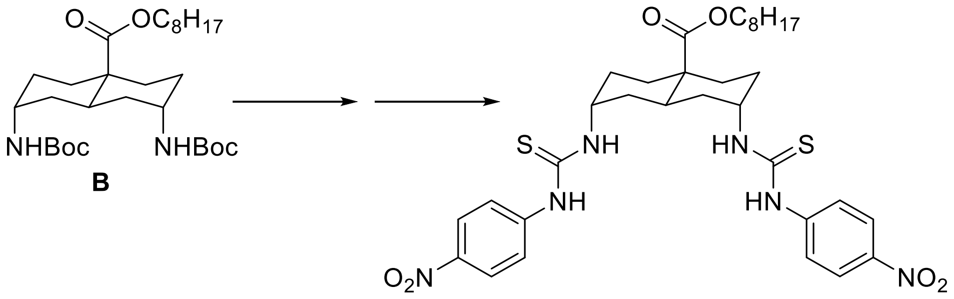

Octyl-ester scaffold **B** (82.7 mg, 158 µmol) was placed in a 25 mL round bottom flask under nitrogen and dry dichloromethane (6 mL) was added. The resulting solution was cooled with an ice bath and trifluoroacetic acid (1.5 mL, 20 mmol, 128 eqv.) was added drop wise. The reaction mixture was stirred at 0 °C for 30 min. and at room temperature for 17 hours. The solvent and trifluoroacetic acid were removed by coevaporation with toluene and methanol. The residue was combined with DMAP (51.9 mg, 463 µmol, 2.9 eqv.), 4-nitrophenyl isothiocyanate (62.8 mg, 349 µmol, 2.2 eqv.), dry THF (15 mL), and DIPEA (1.5 mL, 9.1 mmol, 58 eqv.) and the reaction mixture was stirred for 24 hours at room temperature. The solvent was evaporated and the crude material was redissolved in ethyl acetate (150 mL), washed with aqueous sulphuric acid (0.25 M, 50 mL) and saturated $NaHCO_3$ (50 mL), dried over $MgSO_4$,

filtered and concentrated to dryness. The resulting material was purified by column chromatography over silica gel eluted with 3% methanol in dichloromethane, yielding 51.2 mg (75 µmol, 47%) of transporter **13** as a yellow solid. R_f = 0.23 (3% MeOH in DCM). ^{1}H NMR (500 MHz, acetone-d_6) δ = 9.22 (bs, 2H), 8.18 (d, *J* = 9.2 Hz, 4H), 7.93 (d, *J* = 9.2 Hz, 4H), 7.70 (bs, 2H), 4.59 (bs, 2H), 4.16 (t, *J* = 6.5 Hz, 2H), 2.25 (td, *J* = 13.7, 4.3 Hz, 2H), 2.04 – 1.86 (m, 5H), 1.75 (d, *J* = 14.2 Hz, 2H), 1.74 – 1.64 (m, 2H), 1.63 – 1.50 (m, 4H), 1.46 – 1.24 (m, 10H), 0.88 (t, *J* = 6.6 Hz, 3H). ^{13}C NMR (126 MHz, acetone-d_6) δ = 180.95, 174.86, 147.08, 143.65, 125.22, 121.43, 64.87, 50.58, 48.57, 35.68, 33.57, 33.27, 32.51, 29.94, 29.39, 27.61, 26.81, 23.30, 14.37. ν_{max} (neat)/ cm^{-1}: 3354, 3311, 2926, 2858, 1718, 1596, 1518, 1495, 1315, 1256, 1106, 991, 845, 711. HRMS (ESI): m/z calculated for $C_{33}H_{45}N_6O_6S_2^+$ $[M + H]^+$: 685.2837, found 685.2807.

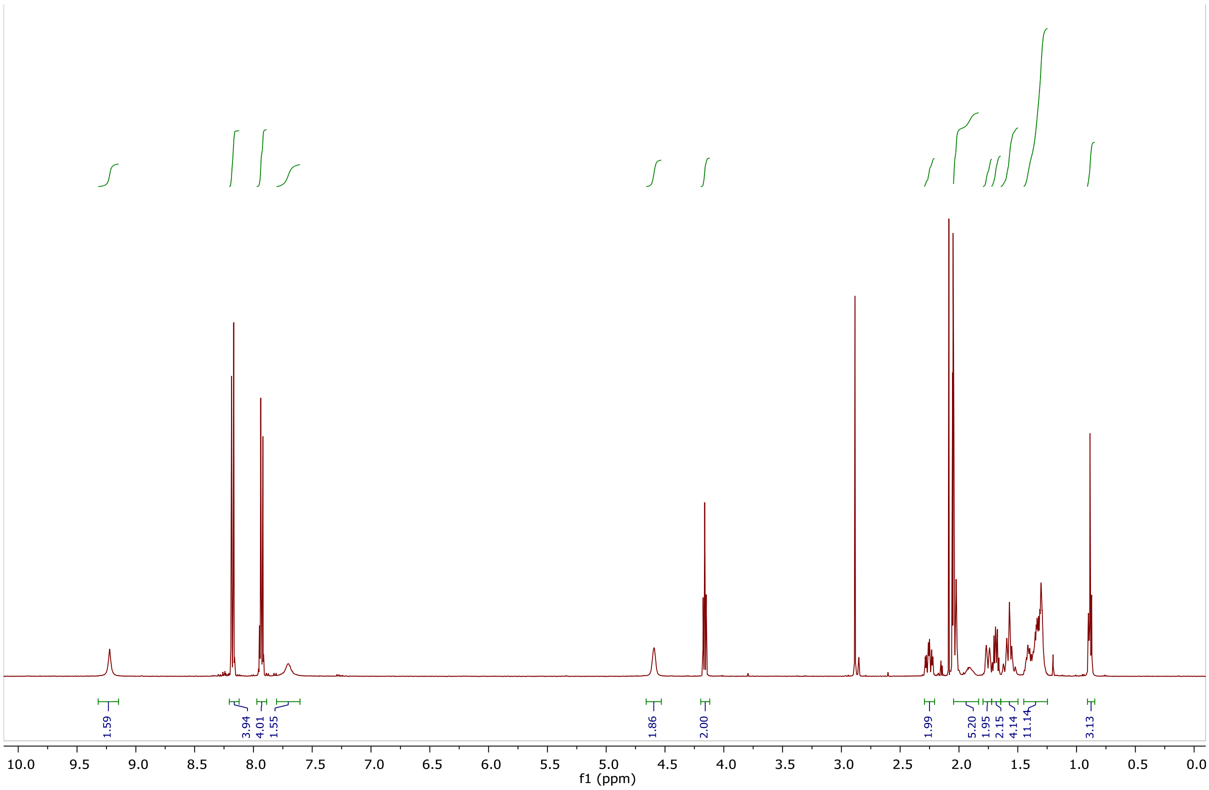

Figure S5. ^{1}H NMR spectrum of **13** in acetone-d$_6$.

2. Binding studies

Measurement of affinities to $Et_4N^+Cl^-$ in chloroform through extraction from water

This method is adapted from Clare *et al.*.[4] The $Et_4N^+Cl^-$ salt was obtained from Sigma Aldrich and dried under vacuum overnight prior to solution preparation using deionised water that had been passed through a Millipore filtration system. All host solutions were prepared using chloroform that had been deacidified by passage through a flash chromatography column containing basic alumina.

A solution of host in deacidified chloroform was combined with 10 mL of aqueous $Et_4N^+Cl^-$ guest solution in a sample tube (30 mL). Concentrations and volumes are specified in Table S1. A magnetic stirring bar (2 cm) was added to the tube and the lower half was immersed in a water bath heated to 303 K, while the contents were stirred vigorously to achieve good mixing of the two phases. After 30 minutes, stirring was stopped and the two phases were allowed to separate. The majority of the aqueous phase was then removed using a pipette and the remaining organic phase was centrifuged for 10 minutes at 4000 rpm. The chloroform solution was then filtered through Whatman 1PS hydrophobic filter paper to remove any remaining aqueous phase. Solvent from the filtrate was then removed *in vacuo* and the solid was further dried under vacuum. The resulting solids from the extraction experiments were dissolved in acetone-d_6 and the solutions were analysed with ^{1}H NMR spectroscopy.

A value for the molar ratio (R) was determined by integrating the signal at $\delta = 3.5$ ppm (C$\underline{H}_2$ of $Et_4N^+Cl^-$) against one of the receptor signals.

The values for R obtained from the ^{1}H NMR spectra were used to calculate values for the equilibrium extraction constants K_e:

$$H_{org} + X^-_{aq} + Y^+_{aq} \overset{K_e}{\rightleftharpoons} HX^-Y^+_{org} \qquad K_e = \frac{[HX^-Y^+]_{org}}{[H]_{org}[X^-]_{aq}[Y^+]_{aq}}$$

$$K_e = \frac{R}{(1-R)\left([G]^{initial}_{aq} - R\frac{V_{org}}{V_{aq}}[H]^{initial}_{org}\right)^2}$$

To convert the extraction constants K_e into association constants K_a it is necessary to obtain the distribution constants K_d for the substrates between aqueous and organic phases:

$$X^-_{aq} + Y^+_{aq} \overset{K_d}{\rightleftharpoons} X^-Y^+_{org} \qquad K_d = \frac{[X^-Y^+]_{org}}{[X^-]_{aq}[Y^+]_{aq}}$$

The K_d value for tetraethylammonium chloride partitioning between water and chloroform has been determined and reported previously to be 1.269×10^{-5} M^{-1}.[5]

The binding constant K_a is then calculated from K_e and K_d:

$$H_{org} + X^{-}Y^{+}_{org} \overset{K_a}{\rightleftharpoons} HX^{-}Y^{+}_{org} \qquad K_a = \frac{[HXY]_{org}}{[H]_{org}[X^{-}Y^{+}]_{org}} = \frac{K_e}{K_d}$$

When [G] > 100 mM, the amount of salt that migrates from the aqueous phase into the organic phase without assistance of the host has to be taken into account by correcting the measured value of R as follows:

$$R = R_{measured} - \frac{K_d\ ([G]_{aq}^{initial})^2}{[H]_{org}^{initial}}$$

Table S1. Overview of the conditions and results from the extraction experiments

Receptor	[H] (mM)	[G] (mM)	V_{org} (mL)	V_{aq} (mL)	$R_{measured}$	K_a (M^{-1})
**9**	0.4	10	3	10	0.17	1.6×10^8
	0.4	15	3	10	0.28	1.4×10^8
	0.4	20	3	10	0.36	1.1×10^8
					**Average:**	$\mathbf{1.4 \times 10^8}$
**10**	0.3	5	3	10	0.27	2.9×10^8
	0.3	10	3	10	0.43	2.6×10^8
	0.3	15	3	10	0.52	2.1×10^8
					**Average:**	$\mathbf{2.5 \times 10^8}$
**13**	0.1	6	8	10	0.46	1.9×10^9
	0.1	8	8	10	0.53	1.4×10^9
	0.1	10	8	10	0.60	1.2×10^9
					**Average:**	$\mathbf{1.5 \times 10^9}$
**17**	0.3	250	3	10	0.30	5.4×10^5
	0.3	400	3	10	0.51	5.0×10^5
					**Average:**	$\mathbf{5.2 \times 10^5}$
**18**	0.3	50	3	10	0.36	1.8×10^7
	0.3	75	3	10	0.51	1.5×10^7
	0.3	100	3	10	0.62	1.3×10^7
					**Average:**	$\mathbf{1.5 \times 10^7}$
**19**	0.3	6	3	10	0.41	1.5×10^9
	0.3	10	3	10	0.66	1.5×10^9
	0.3	20	3	10	0.88	1.4×10^9
					**Average:**	$\mathbf{1.5 \times 10^9}$

Measurement of affinity of **12** to $Bu_4N^+Cl^-$ in DMSO-d_6/H_2O (200:1)

The binding constant to chloride was assessed using ^1H NMR titrations with n-$Bu_4N^+Cl^-$ in DMSO-d_6/H_2O (200:1). The hygroscopic guest n-$Bu_4N^+Cl^-$ and the host compounds were dried under high vacuum to remove residual solvents or water prior to solution preparation. The concentration of the host was around 1 mM in all titrations; the concentration of guest and the volumes of the aliquots of guest solution added to the host solution were varied over the experiments. The guest solutions contained the same concentrations of host as the starting host solution (*i.e.*, n-$Bu_4N^+Cl^-$ was dissolved in a solution of ~1 mM host in DMSO-d_6/H_2O (200:1)), so that the concentration of host did not decrease over the course of the experiment. All ^1H NMR titration binding studies were performed using a Varian 500b NMR spectrometer at 298 K. The shift of both NH signals in DMSO were fitted to 1:1 binding model using a least-squares fitting procedure in a custom-made Excel spreadsheet.

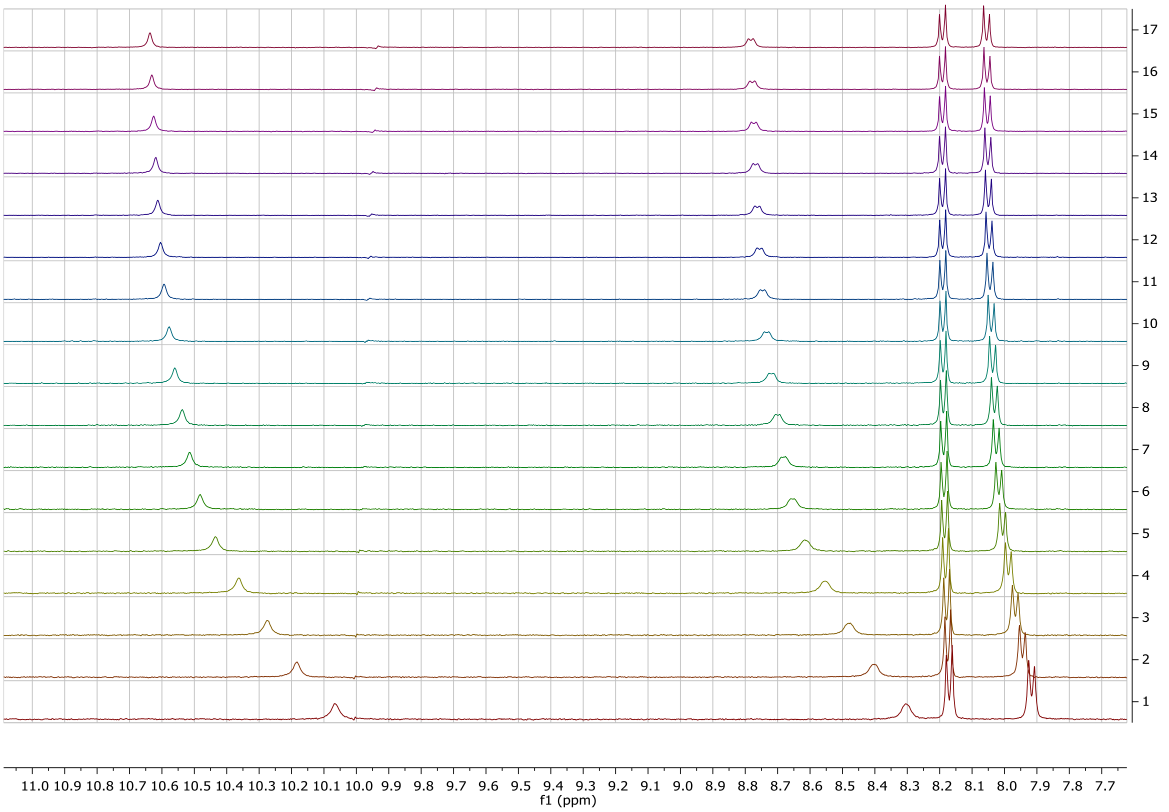

Figure S6. Downfield region of the ^1H NMR spectra from the titration of tetrabutylammonium chloride into **12** in DMSO-d_6/H_2O (200:1) at 298 K.

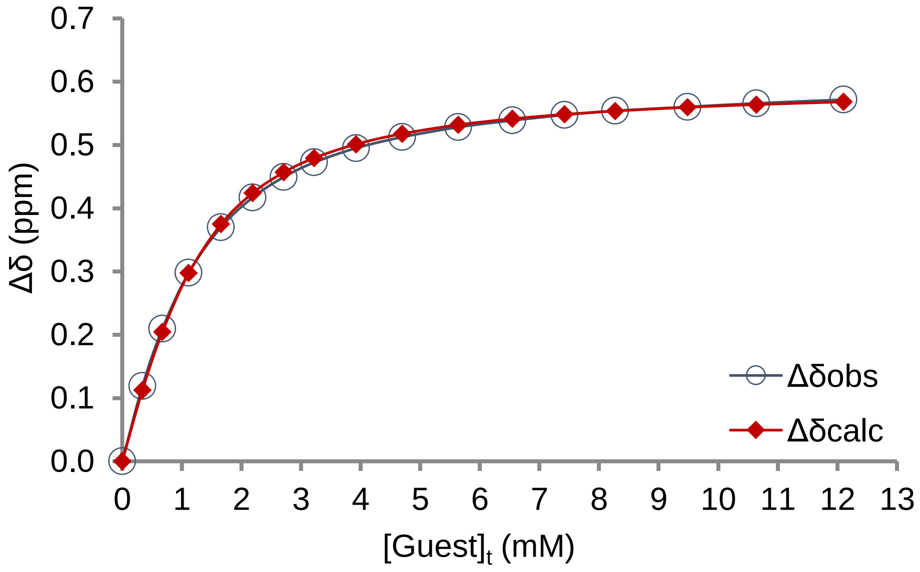

Figure S7. Relationship between the observed binding curve (NH, starting at 10.07 ppm) and calculated fitting for anionophore **12** when titrated against $Bu_4N^+Cl^-$ at 298 K. The movements of both NH signals were used to find K_a = 1719 ± 31 M^{-1}.

3. Transport studies in vesicles

Transport measurements in vesicles with preincorporated anionophores.

1-Palmitoyl-2-oleoyl-*sn*-glycero-3-phosphocholine (POPC) was obtained from Avanti® Polar Lipids, Inc. An extrusion apparatus and 200 nm polycarbonate membranes were obtained from GC Technology Ltd. All lipid and receptor solutions were prepared using chloroform that had been deacidified by passage through a flash chromatography column containing basic alumina, and all aqueous solutions were prepared using deionised water that had been passed through a Millipore filtration system.

POPC and cholesterol solutions (~10 mM) in deacidified chloroform were combined with a solution of transporter in methanol or deacidified chloroform in a 5 mL round bottomed flask. Volumes of the aliquots were calculated from the concentrations of the lipid solution to obtain a POPC to cholesterol ratio of 7:3 (for instance by combining 4.20 μmol POPC and 1.80 μmol cholesterol). The ratio of anionophore to lipid (POPC + cholesterol) was either 1:2500 or 1:25000, as specified in the experiments. The solvents from the lipid/receptor mixture were evaporated under a stream of N_2 and the lipid film was dried under high vacuum for 1 h. The residue was hydrated with 500 μL of an aqueous solution of 10,10'-dimethyl-9,9'-biacridinium nitrate (lucigenin, 0.8 mM) and $NaNO_3$ (225 mM), sonicated for 30 s and stirred for 1 h to give heterogeneous vesicles. Multilamellar vesicles were disrupted by 10 freeze-thaw cycles and then the solution was diluted to 1 mL (by adding 0.5 mL of 225 mM $NaNO_3$) and carefully extruded (29 times) through a polycarbonate membrane (200 nm pore size) to give a uniform distribution of LUVs with an average diameter of ~200 nm.[2] The external lucigenin was removed by passing the solution though a size exclusion column (containing ~2 g Sephadex 50G, eluted with 225 mM $NaNO_3$). The collected vesicles were further diluted with $NaNO_3$ solution (225 mM) to obtain a vesicle solution with 0.4 mM lipid concentration. 3.00 mL of this vesicle solution was placed in a quartz cuvette with a small stir bar and the fluorescence intensity (excitation at 450 nm, emission at 535 nm) was measured over time at 25 ˚C, using a PerkinElmer LS45 fluorescence spectrometer. 75 μL of aqueous NaCl (1.0 M in 225 mM $NaNO_3$, to give an overall external chloride concentration of 25 mM) was added ~30 seconds after the start of the experiment and the fluorescence intensity was measured for another 14 minutes.

Fluorescence data were collected for at least four replicate experiments. The plateau (before addition of chloride) and the vertical drop (the first 1-2.5 seconds after chloride addition, due to quenching of external lucigenin) were removed. Next the data were normalized: all fluorescence values (F) were divided by the fluorescence value before addition of chloride (F_0).

Initial rates (I) were obtained from fluorescence-decay curves (0-500 s) by fitting their inverse (F_0/F) to a double exponential decay function:

$$\frac{F_0}{F} = y - ae^{-bt} - ce^{-dt}$$

Differentiating this gives $\frac{d(\frac{F_0}{F})}{dt} = abe^{-bt} + cde^{-dt}$

and substituting $t = 0$ gives the following equation for initial rate: $I = ab + cd$

Specific initial rates [I] were obtained by dividing the initial rates by the anionophore to lipid ratio and averaging the results over the various ratios measured.[2] These specific initial rates are thus independent of the anionophore to lipid ratio; they are presented in Table 1 of the main text.

Deliverability of anionophores to vesicles.

The vesicles for deliverability studies were prepared as described above for vesicles with preincorporated anionophores, except that anionophores were not added to the initial lipid mixture. After the final dilution, the procedure results in a suspension of vesicles with a notional lipid concentration of 0.4 mM, based on the amounts of POPC and cholesterol added. However, it should be noted that unknown quantities of lipid are lost during the extrusion process, so that the actual concentration is somewhat lower. For external delivery of transporter, this factor affects the final transporter:lipid ratio as discussed below.

A portion of the above vesicle suspension (3 mL) was placed in a quartz cuvette with a small stir bar, which was placed inside the sample compartment of a PerkinElmer LS45 fluorescence spectrometer (at 25 ˚C, stirrer set at maximum speed). Solutions of anionophores were prepared in methanol (HPLC grade) at 96 µM (for experiments at anionophore to lipid ratio 1:2500) or at 9.6 µM (for experiments at 1:25,000). Anionophore solution (5 µL) was then added to the vesicle suspension in the cuvette, employing a 10 µL syringe with rapid plunger action and with

the tip of the syringe needle positioned just above the stir bar. Assuming the notional lipid concentration of 0.4 mM, this would give a transporter to lipid ratio of 1:2500 for **4**, and **5**, **7** or 1:25,000 for the more active anionophores **6**, **8**, **12-16** and **19**. Aqueous NaCl (75 µL, 1.0 M in 225 mM $NaNO_3$, to give an overall external chloride concentration of 25 mM Cl^-) was added 5 minutes after anionophore addition and ~30 seconds after the start of fluorescence measurements.

Fluorescence data for external delivery experiments were processed in an identical manner as transport studies using vesicles with preincorporated anionophores. Initial rates were obtained from double exponential fits to the inverse of the F/F_0 curves (0-500 s) as described above. Deliverability was assessed by dividing the initial rate from external addition of anionophores by the initial rate from transport experiments using preincorporated anionophores, to give a deliverability index *D*.

For the most deliverable compounds *D* was found to be >1. We presume this results from the fact that, due to losses during vesicle preparation, the actual concentration of lipid is less than the notional value of 0.4 mM. Addition of transporter after vesicle preparation, according to the procedure above, thus gives transporter:lipid ratios which are higher than intended. By contrast, for preincorporated transporters, both lipid and transporter are lost together so that the ratios are as intended. A perfectly deliverable anionophore should thus appear less active when preincorporated than when added to preformed vesicles. The results are illustrated in Figures S8 – S14 below, which show traces corresponding to external addition (broken lines) and preincorporation (solid lines) for each anionophore.

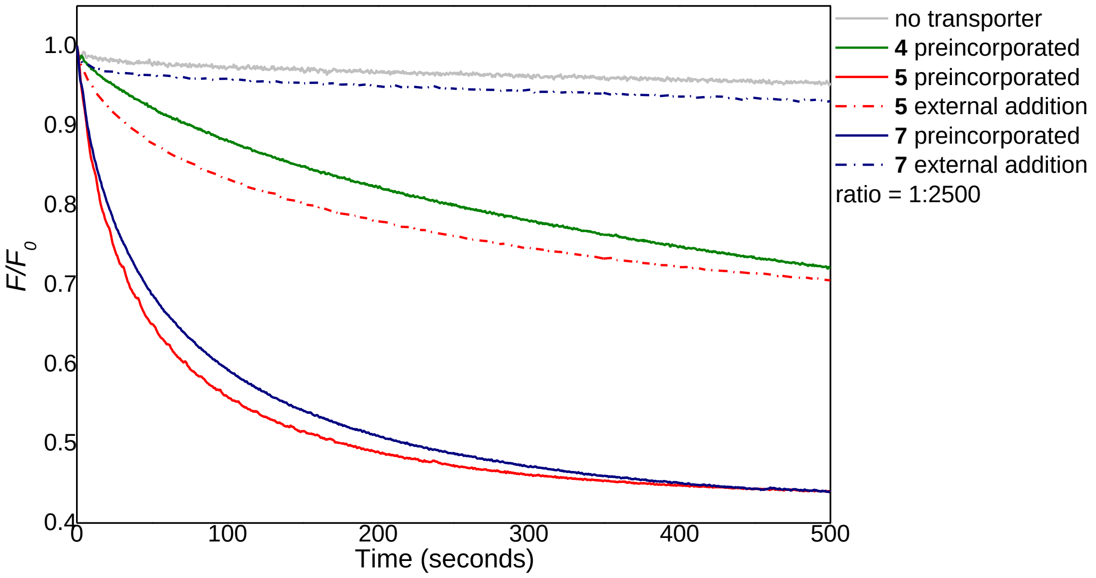

Figure S8. Chloride transport by compounds **4**, **5** and **7** into 200 nm POPC/cholesterol (7:3) vesicles when anionophores are either preincorporated into the membrane (continuous lines) or added externally as a solution in methanol to vesicles without anionophores (dashed dotted lines). The notional transporter:lipid ratio is 1:2500 in all experiments.

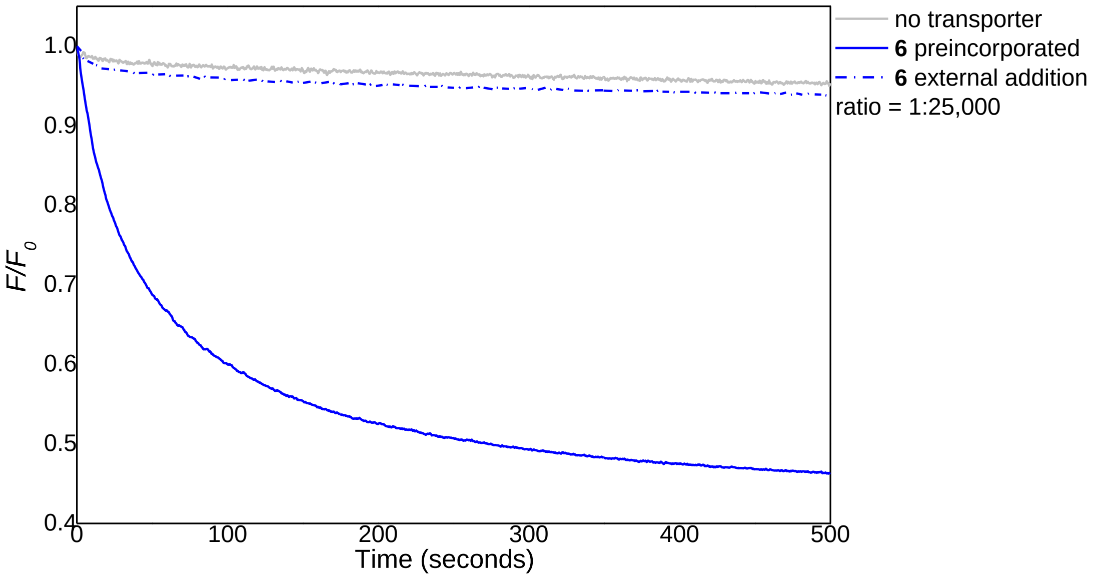

Figure S9. Chloride transport by compound **6** into 200 nm POPC/cholesterol (7:3) vesicles when anionophores are either preincorporated into the membrane (continuous lines) or added externally as a solution in methanol to vesicles without anionophores (dashed dotted lines). The notional transporter:lipid ratio is 1:25,000 in all experiments.

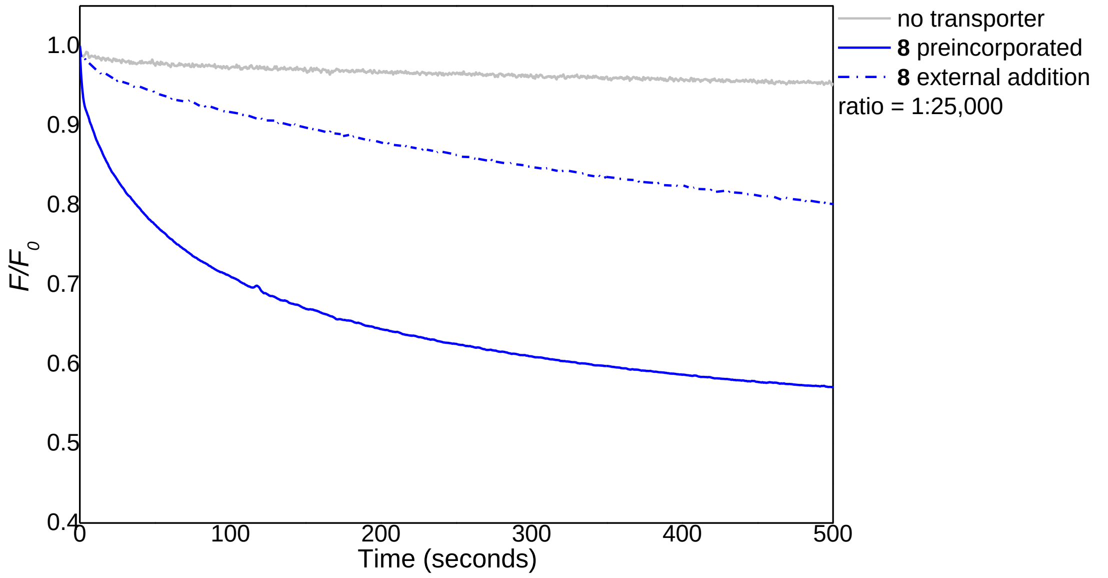

Figure S10. Chloride transport by compound **8** into 200 nm POPC/cholesterol (7:3) vesicles when anionophores are either preincorporated into the membrane (continuous lines) or added externally as a solution in methanol to vesicles without anionophores (dashed dotted lines). The notional transporter:lipid ratio is 1:25,000 in all experiments.

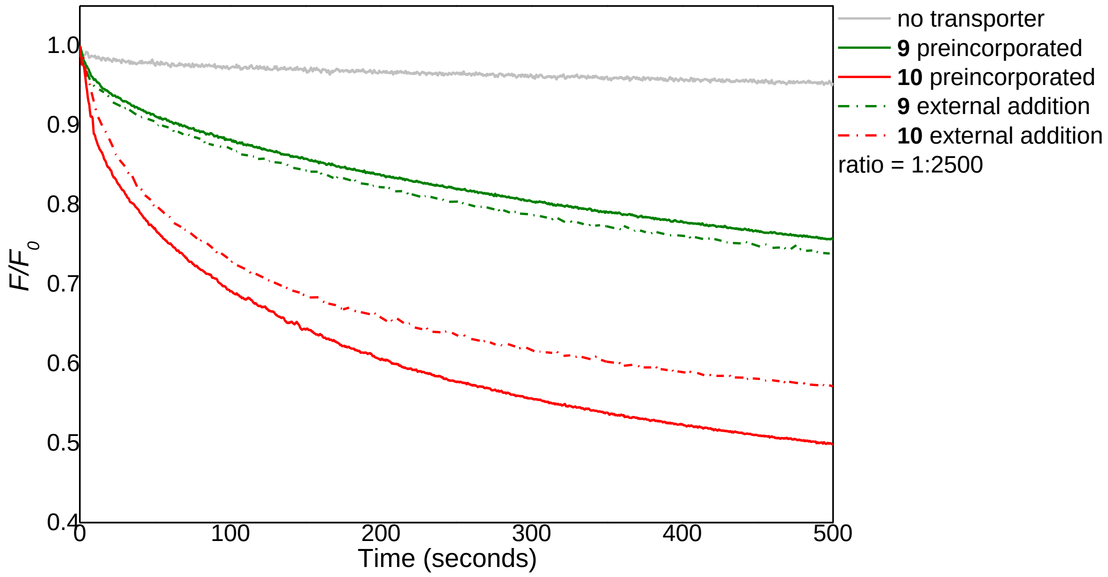

Figure S11. Chloride transport by compounds **9** and **10** into 200 nm POPC/cholesterol (7:3) vesicles when anionophores are either preincorporated into the membrane (continuous lines) or added externally as a solution in methanol to vesicles without anionophores (dashed dotted lines). The notional transporter:lipid ratio is 1:2500 in all experiments.

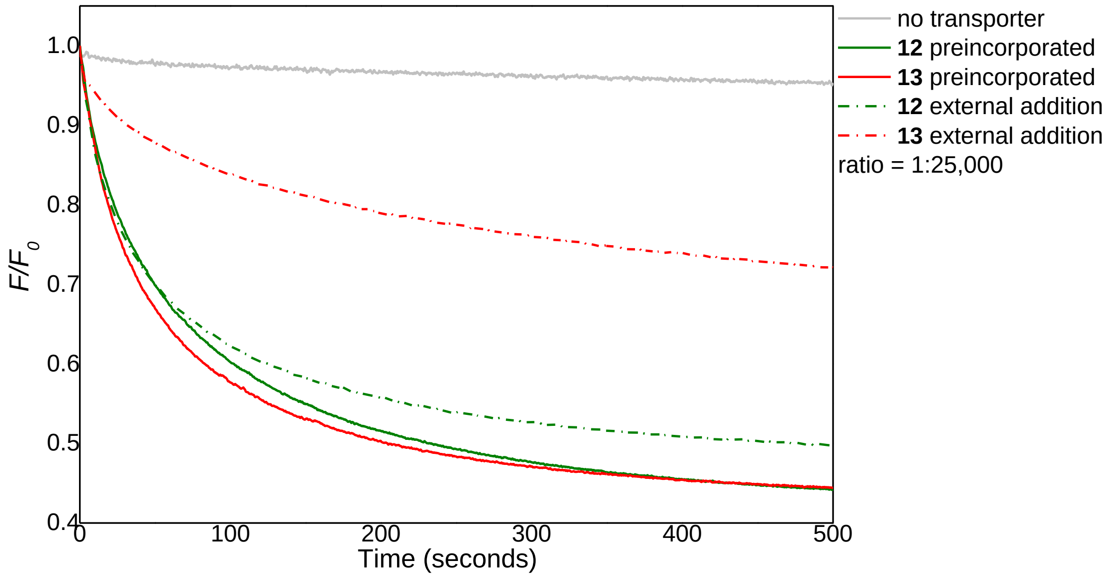

Figure S12. Chloride transport by compounds **12** and **13** into 200 nm POPC/cholesterol (7:3) vesicles when anionophores are either preincorporated into the membrane (continuous lines) or added externally as a solution in methanol to vesicles without anionophores (dashed dotted lines). The notional transporter:lipid ratio is 1:25,000 in all experiments.

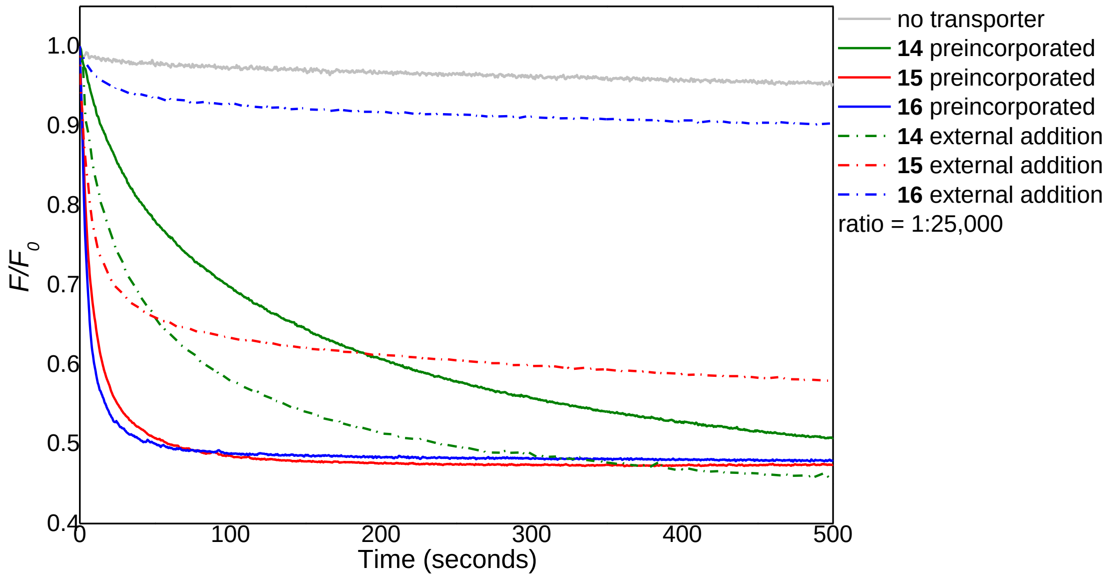

Figure S13. Chloride transport by compounds **14**–**16** into 200 nm POPC/cholesterol (7:3) vesicles when anionophores are either preincorporated into the membrane (continuous lines) or added externally as a solution in methanol to vesicles without anionophores (dashed dotted lines). The notional transporter:lipid ratio is 1:25,000 in all experiments.

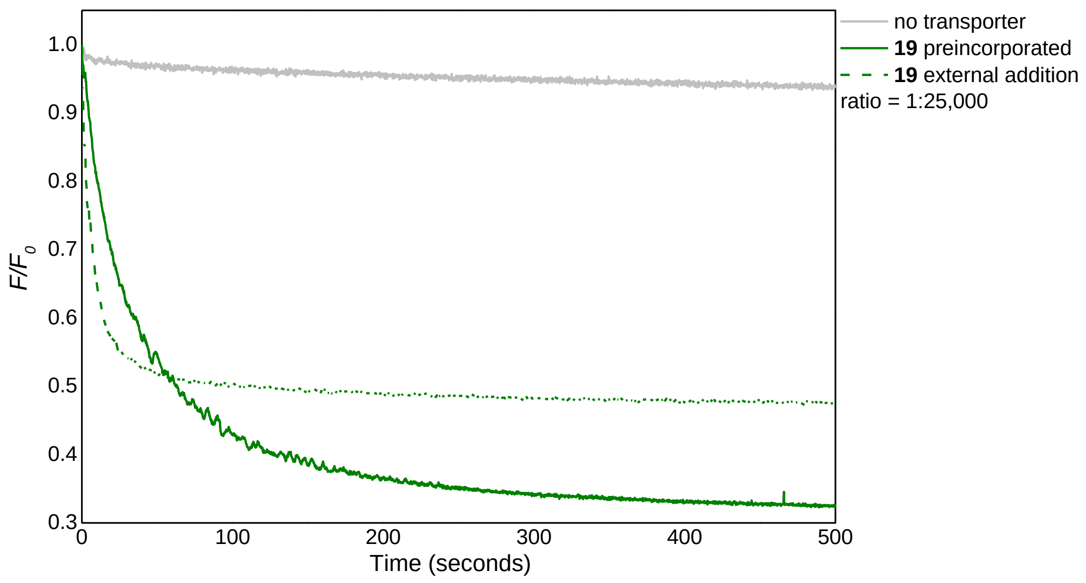

Figure S14. Chloride transport by compound **19** into 200 nm POPC/cholesterol (7:3) vesicles when anionophores are either preincorporated into the membrane (continuous lines) or added externally as a solution in methanol to vesicles without anionophores (dashed dotted lines). The notional transporter:lipid ratio is 1:25,000 in all experiments.

4. Biological Data

Materials and Methods

Cells and cell culture

FRT cells stably expressing the halide sensor YFP-H148Q/I152L[6] were generous gifts of AS Verkman (University of California, San Francisco), while CFBE41o$^-$ cells[7] stably expressing YFP (YFP-CFBE cells) were a generous gift of LJV Galietta (Telethon Institute for Genetics and Medicine). YFP-FRT cells were cultured as described previously for FRT cells[8] except that media contained 10% fetal bovine serum, 2 mM L-glutamine and the selection agent G418 (YFP-FRT cells, 0.5 mg ml^{-1}; WT-CFTR-YFP-FRT cells, 0.75 mg ml^{-1}) or zeocin (F508del-CFTR-YFP-FRT cells, 0.5 mg ml^{-1}). YFP-CFBE cells were cultured in minimal essential medium (MEM) supplemented with 10% fetal bovine serum, 2 mM L-glutamine, 100 U ml^{-1} penicillin and 100 mg ml^{-1} streptomycin. All cells were incubated at 37 °C in a humidified atmosphere with 5% CO_2.

For fluorescence microplate reader studies, cells were seeded in 96-well plates and grown in culture media at 37 °C in a humidified atmosphere with 5% CO_2 for 3 – 4 days until 90% confluent. To promote the plasma membrane expression of F508del-CFTR, prior to study F508del-YFP-FRT cells were either cultured at 27 °C for 24 h or treated with lumacaftor (3 μM) in YFP-FRT media containing 1% fetal bovine serum at 37 °C for 24 h. For fluorescence microscopy studies, YFP-FRT cells were plated on glass coverslips and used 4 – 5 days later when cell confluency was 80%.

Fluorescence plate reader assay

Anionophore-mediated anion transport was quantified by measuring I$^-$-induced quenching of YFP fluorescence at 37 °C using a microplate reader following the method of Galietta et al..[9] The microplate reader (model: FLUORstar Omega; BMG LABTECH Ltd., Aylesbury, UK) was equipped with a syringe pump and excitation (500 ± 10 nm) and emission (535 ± 15 nm) filters (Chroma Technology GmbH, Olching, Germany). After washing cells twice with PBS (composition (mM): 137 NaCl, 2.7 KCl, 8.1 Na_2HPO_4, 1.5 KH_2PO_4, 1 $CaCl_2$ and 0.5 $MgCl_2$, pH 7.40), cells were incubated with test anionophores (1 – 50 μM) or the vehicle (DMSO [0.1 – 0.5% v v^{-1}]) in 40 μl of PBS for 10 – 120 minutes according to the experimental design. Other compounds, including forskolin, ivacaftor and channel inhibitors were added to

the PBS bathing cells 10 minutes prior to PBS iodide addition, whereas UTP was added to cells with the PBS iodide solution. Fluorescence was measured for 14 seconds; 2 seconds before and 12 seconds after injection of 110 μl PBS iodide solution (137 mM Cl^- replaced by I^-) into each well (to obtain a final concentration of I^- of 100 mM).

We expressed cell fluorescence values (F) relative to the fluorescence value immediately before I^- injection (F_0), excluding the initial decrease, which likely represents the quenching of extracellular YFP released from cells and signal reduction accompanying the increase in solution volume bathing cells. To quantify anion transport by test anionophores, we determined the initial slope of the fluorescence decay following I^- addition by fitting first order exponential functions to the last 12 seconds of each fluorescence recording. In each experiment, n values represent the mean of two replicates and all treatments were tested at least three times using different microplates.

Fluorescence microscopy

To investigate the duration of anionophore-mediated anion transport, we used fluorescence microscopy as described previously.[10] In brief, YFP-FRT cells were washed 3x with PBS before transfer to a perfusion chamber mounted on the stage of a Leica DM IRB microscope. YFP-FRT cells were treated with anionophore (10 μM) in DMSO (0.1% v v^{-1}) in 1 ml PBS for 10, 60 or 120 minutes at 37 °C. Any anionophore not incorporated into YFP-FRT cell membranes was removed from the chamber by perfusion with PBS when fluorescence measurements commenced. For fluorescence measurements, a field of view with bright YFP-FRT cells was selected and the cells were perfused with (i) PBS for 5 minutes then (ii) PBS containing I^- (10 mM) (made by preparing PBS with 127 mM NaCl to maintain osmolarity) for 5 minutes and finally (iii) PBS for 30 minutes to remove thoroughly I^- from the perfusion chamber. YFP-FRT cells were repeatedly treated with I^- (10 mM) and then washed with PBS to assess the duration of anionophore-mediated anion transport. During all interventions, the rate of solution perfusion was 8 – 10 ml min^{-1}; temperature was 37 °C and except for the first 15 minutes of PBS washing, no data were collected between cycles of I^- perfusion and PBS washing.

For cell fluorescence measurements, we used the Volocity (Improvision) data acquisition and analysis system and a cooled CCD camera (Hamamatsu ORCA ER firewall) with the Leica DM IRB inverted fluorescence microscope equipped with an oil objective (x65,

numerical aperture 1.32), excitation filter wheel and multiple band dichroic and emission filters (YFP: excitation, 500 ± 10 nm; emission, 545 ± 25 nm). Cell fluorescence data were sampled every 6 seconds.

Fluorescence data from 5 – 9 cells per coverslip were analysed with 4 coverslips tested for each anionophore. Cell fluorescence values (F) are expressed relative to the fluorescence value immediately before I^- (10 mM) addition to YFP-FRT cells (F_0). By fitting exponential functions to the first 2 minutes of the fluorescence decay following I^- (10 mM) addition, we determined the initial slope to quantify anion transport by test anionophores.

Cytotoxicity studies

For cytotoxicity studies, we used YFP-CFBE and YFP-FRT cells. YFP-CFBE cells were seeded in 96-well plates at a density of 5 x 10^4 cells/well and cultured at 37 °C in a humidified atmosphere of 5% CO_2. Thirty hours after seeding the cells, different concentrations of anionophores were added to the culture media and the cells were incubated with anionophores for 18 h. To assay cell viability, we used the XTT Cell Proliferation Assay Kit (ATCC) according to the manufacturer's instructions. The specific absorbance of test samples was measured using a Microplate Spectrophotometer (SpectraMax 190, Molecular Devices, Sunnyvale, CA, USA). In each experiment, multiple replicates (12 – 25 wells from 3 – 5 plates) were tested for each concentration of anionophore.

To evaluate the viability of YFP-FRT cells, we used the IncuCyte ZOOM™ (Essen Bioscience (now Sartorius), Welwyn Garden City, UK) live-cell analysis system. YFP-FRT cells were seeded in 96-well plates at a density of 4000 cells well^{-1} and allowed to grow for 48 h at 37 °C in a humidified atmosphere of 5% CO_2. Then, the YFP-FRT culture media was replaced with fresh culture media containing test anionophores or the vehicle (DMSO 0.5% v v^{-1}) and deep red anthraquinone 7 (DRAQ7; Biostatus, Shepshed, UK) was added following the manufacturer's instructions before the YFP-FRT cells were transferred to the IncuCyte ZOOM™. At 2 h intervals over a 24 h period, 4 images of each well on 96-well plates were acquired; the phase contrast images were used to determine the total number of cells per well, while the fluorescence images to identify the number of dead cells with nuclei labelled red by DRAQ7. In each experiment, one data point is the average value from four wells.

To test for the induction of apoptosis by anionophores, we also used the IncuCyte ZOOM™ (Essen Bioscience) live-cell analysis system. YFP-FRT cells were seeded and grown on 96-well plates as described above for cell viability studies. After 48 h, the YFP-FRT culture media was replaced with fresh culture media containing test anionophores or the vehicle (DMSO 0.5% v v^{-1}) and IncuCyte annexin V red reagent (Essen Bioscience) was added following the manufacturer's instructions before the YFP-FRT cells were transferred to the IncuCyte ZOOM™. At 2 h intervals over a 24 h period, 4 images of each well on 96-well plates were acquired; the phase contrast images were used to determine the total number of cells per well, while the fluorescence images to identify the number of apoptotic cells labelled red by the IncuCyte annexin V red reagent. In each experiment, one data point is the average value from three wells.

Reagents

Except for ivacaftor and lumacaftor (Selleck Chemicals; Stratech Scientific Ltd., Newmarket, UK), chemicals were supplied by the Sigma-Aldrich Company Ltd. (now Merck KGaA) (Gillingham, UK). UTP was dissolved in distilled water, forskolin in methanol, while anionophores and channel modulators were prepared in DMSO. Stock solutions were stored at –20 °C except those of anionophores, which were stored at room temperature. Immediately before use, stock solutions were diluted to final concentrations. Precautions against light-sensitive reactions were observed when using anionophores and channel modulators. DMSO is without effect on the activity of CaCC[11] and CFTR.[12]

Additivity of anion transport by anionophores and epithelial Cl^- channels

Figure S15 shows the chemical structures of small molecules used to modulate the activity of the Ca^{2+}-activated Cl^- channel (CaCC). They include the P2Y receptor agonist UTP, which is used to elevate the intracellular Ca^{2+} concentration, leading to the activation of the CaCC and the CaCC inhibitor $CaCC_{inh}$-A01, identified in a high-throughput screen of TMEM16A inhibitors.[13]

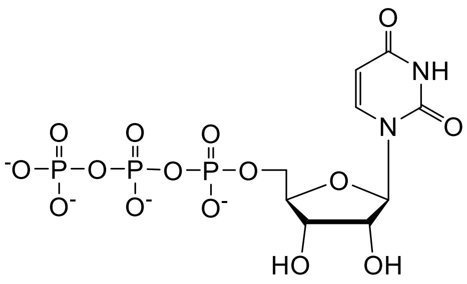

UTP

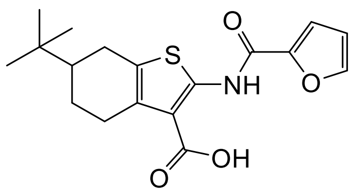

$CaCC_{inh}$-A01

Figure S15. Chemical structures of small molecules used to modulate CaCC activity. UTP elevates the intracellular Ca^{2+} concentration and hence, activates the CaCC. $CaCC_{inh}$-A01 inhibits the CaCC.

Figure S16 shows the relationship between anionophore concentration and anion transport for **11**, **12**, **15** and **19** in YFP-FRT cells. For each anionophore, as the transporter concentration was raised, there was a progressive increase in the rate of fluorescence decay and hence, anion transport activity (Fig. S16). Figure S16 also compares anionophore-concentration response relationships in YFP-FRT and YFP-CFBE cells. For anionophores **11** and **12**, the relationships were identical, for **19**, they were similar, but for **15**, they were different (Fig. S16). The difference in efficacy of **15** between YFP-CFBE and YFP-FRT cells likely relates to its reduced deliverability compared to **11** and **12**.

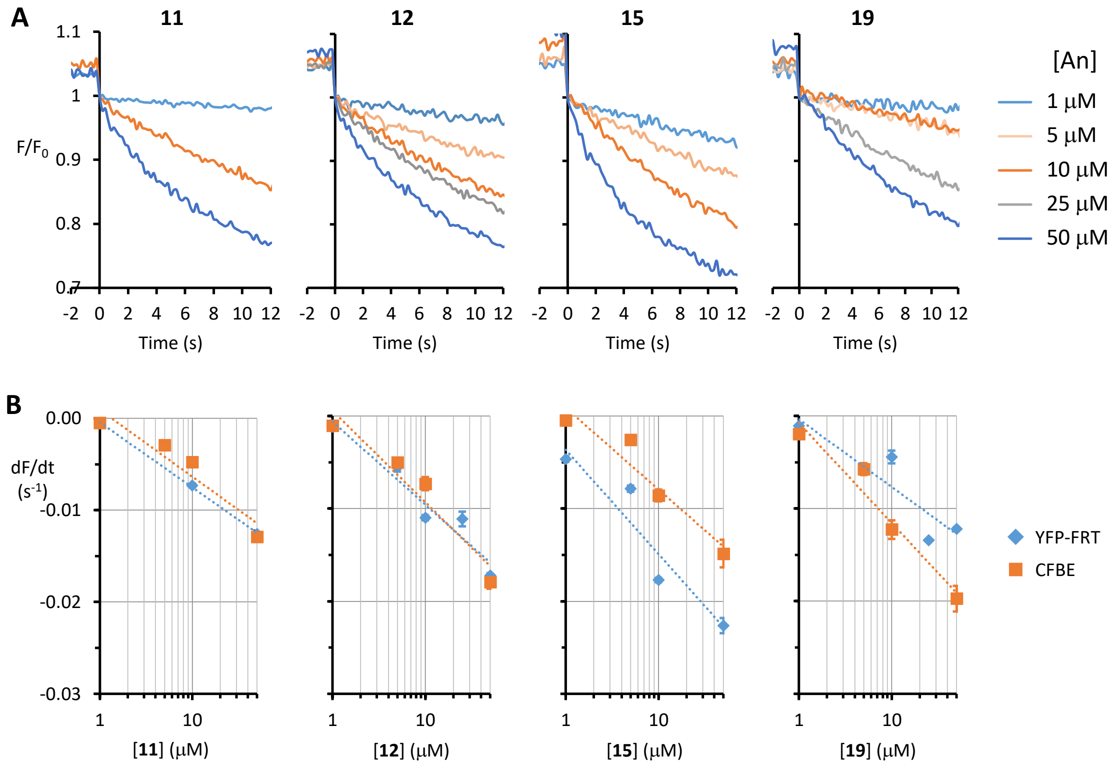

Figure S16. Concentration-dependence of anionophore activity. (**A**) Representative time courses of cell fluorescence from YFP-FRT cells treated with increasing concentrations of anionophores (An) **11**, **12**, **15** and **19** (1 – 50 μM) in DMSO (1 – 10 μM, 0.1% v v^{-1}; 25 and 50 μM, 0.5% v v^{-1}). Cell fluorescence values were normalised to the fluorescence intensity before I^- (100 mM) addition at t = 0 s. (**B**) Relationship between anionophore concentration and anion transport activity for compounds **11**, **12**, **15** and **19** in YFP-FRT and YFP-CFBE cells. Fluorescence quenching by the anionophore vehicle DMSO was subtracted from each test measurement. Data are means ± SEM (n = 25 – 65 from at least four independent experiments); dotted lines show the fit of first-order regression functions to mean data.

To investigate further anion transport by anionophores and CaCCs in YFP-CFBE cells, we used the aroylaminothiophene CaCC inhibitor $CaCC_{inh}$-A01 (see Fig. S15), which fully blocks CaCC in human bronchial epithelial cells.[13] Figure S17A shows representative time courses of fluorescence decay for the anionophores **11**, **12**, **15** and **19** (all tested at 50 μM) in the absence and presence of UTP (1 mM) and $CaCC_{inh}$-A01 (10 μM), while Figure S17B reports summary anion transport data determined from initial slope measurements, averaged over multiple experiments (n = 12 – 45 from at least four independent experiments). Consistent with previous results,[13] $CaCC_{inh}$-A01 inhibited CaCC activity stimulated by UTP (Fig. S17).

CaCC$_{inh}$-A01 (10 μM) also showed a direct inhibitory effect on transport by **19**, although this was not observed for **11**, **12** and **15**.

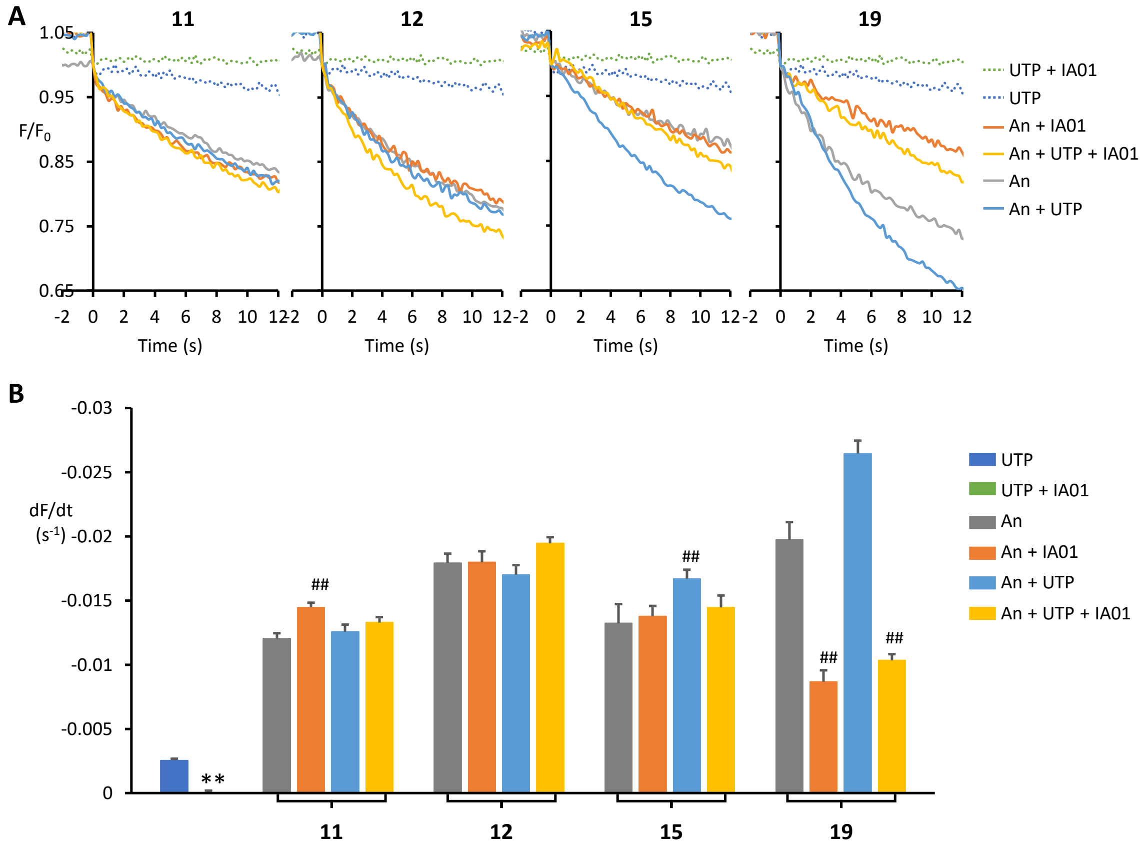

Figure S17. Impact of the CaCC inhibitor CaCC$_{inh}$-A01 on anionophore activity. (**A**) Representative time courses of cell fluorescence from YFP-CFBE cells treated with the anionophores (An) **11**, **12**, **15** and **19** (50 μM) in DMSO (0.5% v v^{-1}), UTP (1 mM) and the CaCC inhibitor CaCC$_{inh}$-A01 (IA01; 10 μM) as indicated. Cell fluorescence values were normalised to the fluorescence intensity before I^- (100 mM) addition at t = 0 s. (**B**) Effects of CaCC$_{inh}$-A01 (10 μM) on anionophore activity. Summary data show anionophore-mediated anion transport for the indicated conditions determined from the initial slope of the fluorescence decay. Fluorescence quenching by the anionophore vehicle (DMSO, 0.5% v v^{-1}) was subtracted from each test measurement. Data are means ± SEM (n = 12 – 45 from at least four independent experiments); **, *P* < 0.01 vs. UTP; $^{\#\#}$, *P* < 0.01 vs. anionophore.

Figure S18 shows the chemical structures of small molecules that modulate CFTR function. The cAMP agonist forskolin activates adenylate cyclase leading to an increase in the intracellular concentration of cAMP. Phosphorylation of CFTR by the cAMP-dependent protein kinase (PKA) is a prerequisite for CFTR activity.[14] The thiazolidinone CFTR$_{inh}$-172 is a widely used inhibitor of the CFTR Cl^- channel.[15] Lumacaftor and ivacaftor are the first clinically-licensed drugs to target CF mutations. Lumacaftor is a CFTR corrector, which traffics F508del-CFTR to the plasma membrane,[16] while ivacaftor is a CFTR potentiator, which enhances greatly F508del-CFTR activity after its phosphorylation by PKA.[17]

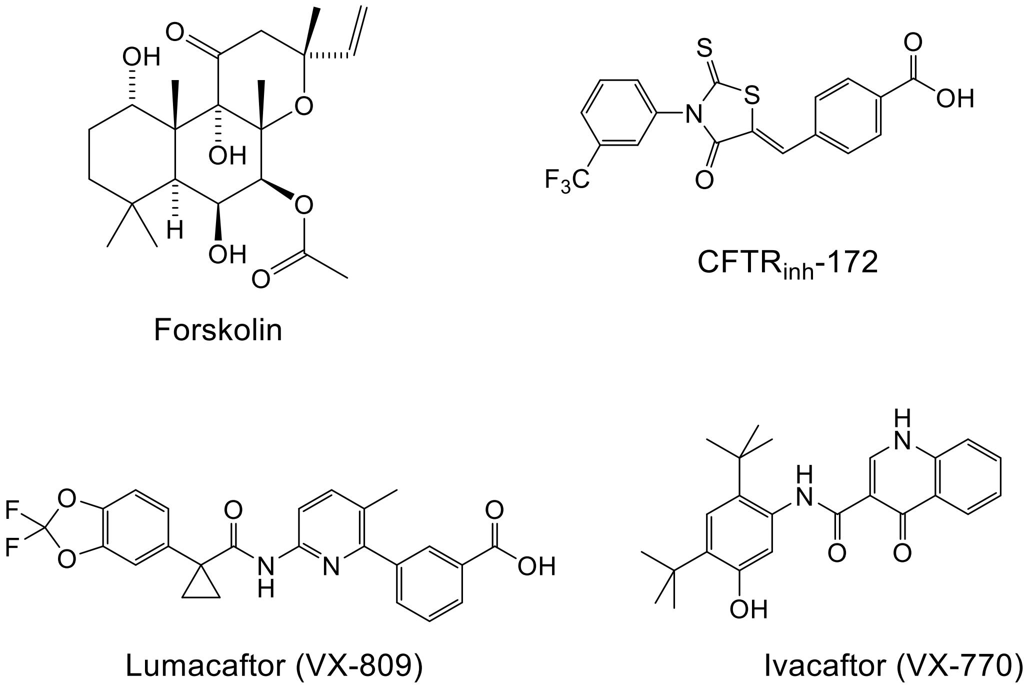

Figure S18. Chemical structures of small molecule CFTR modulators. Forskolin elevates the intracellular concentration of cAMP, while $CFTR_{inh}$-172 inhibits the CFTR Cl^- channel. The clinically-licensed drugs lumacaftor and ivacaftor restore function to mutant CFTR.

Figure S19 investigates whether anion transport by anionophores **11**, **12**, **15** and **19** is additive to that of wild-type CFTR. The cAMP agonist forskolin strongly stimulated anion transport in WT-CFTR-YFP-FRT cells. Of note, anion transport by each anionophore was additive to that of WT-CFTR (Fig. S19). Figure S19 also shows that the CFTR inhibitor $CFTR_{inh}$-172 was without effect on the activity of compounds **11** and **12**, but had some effects on compounds **15** and **19**. For discussion of these data, see the manuscript text.

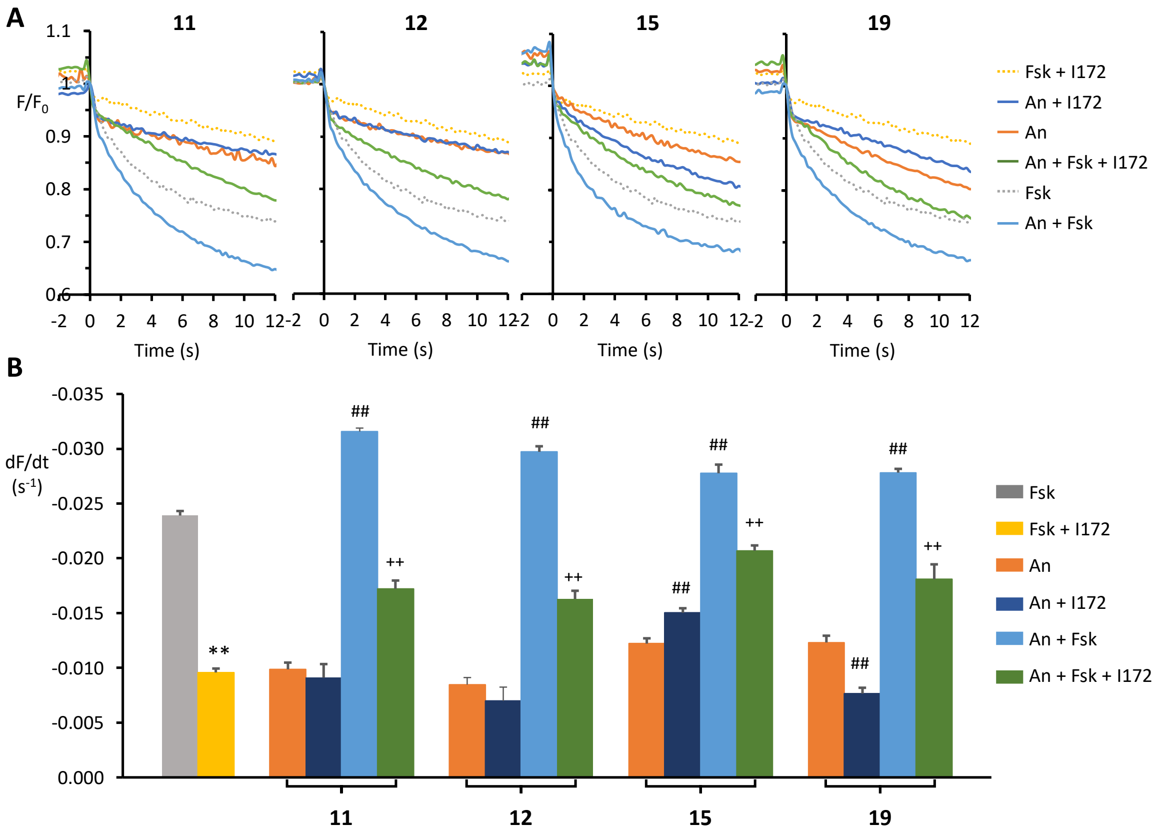

Figure S19. Anion transport by anionophores and CFTR is additive. (**A**) Representative time courses of cell fluorescence from YFP-FRT cells expressing wild-type CFTR treated with the anionophores (An) **11**, **12**, **15** and **19** (50 μM) in DMSO (0.5% v v^{-1}), forskolin (fsk; 10 μM) and the CFTR inhibitor $CFTR_{inh}$-172 (I172; 10 μM) as indicated. Cell fluorescence values were normalised to the fluorescence intensity before I^- (100 mM) addition at t = 0 s. (**B**) Effects of CFTR modulators on anionophore activity. Summary data show anionophore-mediated anion transport for the indicated conditions determined from the initial slope of the fluorescence decay. Fluorescence quenching by the anionophore vehicle (DMSO, 0.5% v v^{-1}) was subtracted from each test measurement. Data are means ± SEM (n = 12 – 72 from at least four independent experiments); **, P < 0.01 vs. forskolin; $^{\#\#}$, P < 0.01 vs. anionophore; $^{++}$, P < 0.01 vs. anionophore + forskolin.

Figure S20 shows representative time courses of fluorescence decay from FRT cells co-expressing the CF mutation F508del and YFP. In control F508del-CFTR-YFP-FRT cells cultured at 37 °C, F508del-CFTR is all, but absent from the plasma membrane and forskolin (10 μM) stimulates little or no fluorescence decay (Fig. S20A). By contrast, when F508del-CFTR-YFP-FRT cells are incubated at 27 °C for 24 h or treated with the CFTR corrector lumacaftor (3 μM for 24 h at 37 °C), F508del-CFTR is delivered to the plasma membrane and forskolin (10 μM) stimulates a decay of fluorescence (Fig. S20A). Under all conditions, treatment of F508del-CFTR-YFP-FRT cells with the CFTR potentiator ivacaftor (1 μM) accelerates the rate of fluorescence decay stimulated by forskolin (10 μM) (Fig. S20B).

Similarly, anion transport by anionophores **11**, **12**, **15** and **19** (50 μM) is additive to rescue of F508del-CFTR irrespective of the method used (Fig. S20).

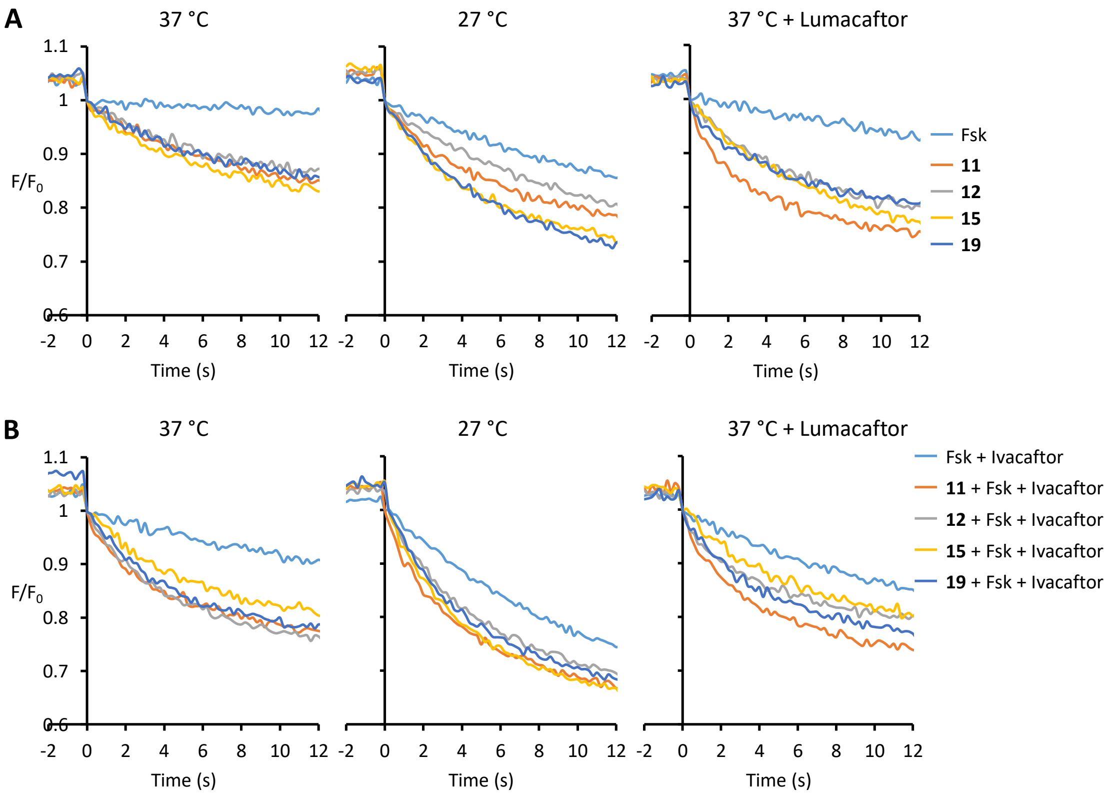

Figure S20. Anion transport by anionophores in YFP-FRT cells expressing F508del-CFTR. (**A**) and (**B**) Representative time courses of cell fluorescence for YFP-FRT cells expressing F508del-CFTR treated with the anionophores **11**, **12**, **15** and **19** (50 μM) in DMSO (0.5% v v^{-1}). Control F508del-CFTR-YFP-FRT cells were cultured at 37 °C. To rescue the plasma membrane expression of F508del-CFTR, cells were either cultured at 27 °C for 24 h or treated with lumacaftor (3 μM for 24 h at 37 °C). In (**A**), anion transport by F508del-CFTR activated with forskolin (fsk; 10 μM) is compared with the action of test anionophores. In (**B**), anion transport by F508del-CFTR activated with forskolin (10 μM) and potentiated with ivacaftor (1 μM) is compared with the action of anionophores together with F508del-CFTR stimulation and potentiation with forskolin and ivacaftor. Cell fluorescence values were normalised to the fluorescence intensity before I^- (100 mM) addition at t = 0 s. For summary data, see Figure 4.

Anionophore delivery, persistence and efficacy

In previous work,[10] we demonstrated that the anionophore **11** remains active at the plasma membrane for >2 h. To investigate further the relationship between anionophore delivery to cell membranes, the longevity of their action and their efficacy, we undertook two types of experiment. First, using the microplate reader, we tested the effects of different

incubation periods on anion transport by **11**, **12**, and **19** (each tested at 1, 10 and 50 μM). Second, using fluorescence microscopy, we examined the persistence of anion transport by the two most promising anionophores, **11** and **12**, (both tested at 10 μM) after different incubation periods. Because of its reduced deliverability, we did not use **15** in these experiments.

Figure S21 demonstrates that the effect of prolonging the incubation period of anionophores with YFP-CFBE cells was both anionophore- and concentration-dependent. The data also show that **12** and **19** are more powerful anionophores than **11**, particularly at longer incubation periods. For **11**, increasing the incubation period from 10 to 120 minutes achieved little improvement in anion transport (Fig. S21B). By contrast, anion transport by **12** and **19** were both concentration- and incubation period-dependent. For **12** (10 μM) and **19** (10 and 50 μM), anion transport increased noticeably from 10 to 120 minutes, but for **12** (50 μM) there was a dramatic acceleration of anion transport at 60 and particularly 120 minutes (Fig. S21B). This latter effect is difficult to explain and merits further investigation.

To investigate the effect of incubation time on the persistence of anion transport, we used fluorescence microscopy, which facilitated cell perfusion. For consistency with previous studies,[10] we used YFP-FRT cells. After treating YFP-FRT cells with **11** and **12** (both at 10 μM) for 10, 60 or 120 minutes at 37 °C, any unincorporated compound was removed by washing with iodide-free PBS for 5 minutes. Then, cells were exposed to I^- (10 mM) for 5 minutes before washing with iodide-free PBS for 30 minutes. Subsequently, the cells were subjected to repeated cycles of I^- (10 mM) addition and washing with PBS. Figure S22A shows the time course of fluorescence for cells incubated with **11** and **12** for 10, 60 and 120 minutes. All I^- (10 mM) addition/PBS washing cycles produced decreases in fluorescence followed by recovery (Fig. S22A), demonstrating the persistence of anion transport by anionophores **11** and

12. Analysis of the effect of increasing the incubation period revealed a small positive effect on anion transport by the compounds (Fig. S22B). Several conclusions can be drawn from the data in Figures S21 and S22. First, anion transport by anionophores is concentration-dependent. Second, increasing the incubation period improves anion transport by some, but not all anionophores. Third, for at least two anionophores, **11** and **12**, their effects show noticeable persistence.

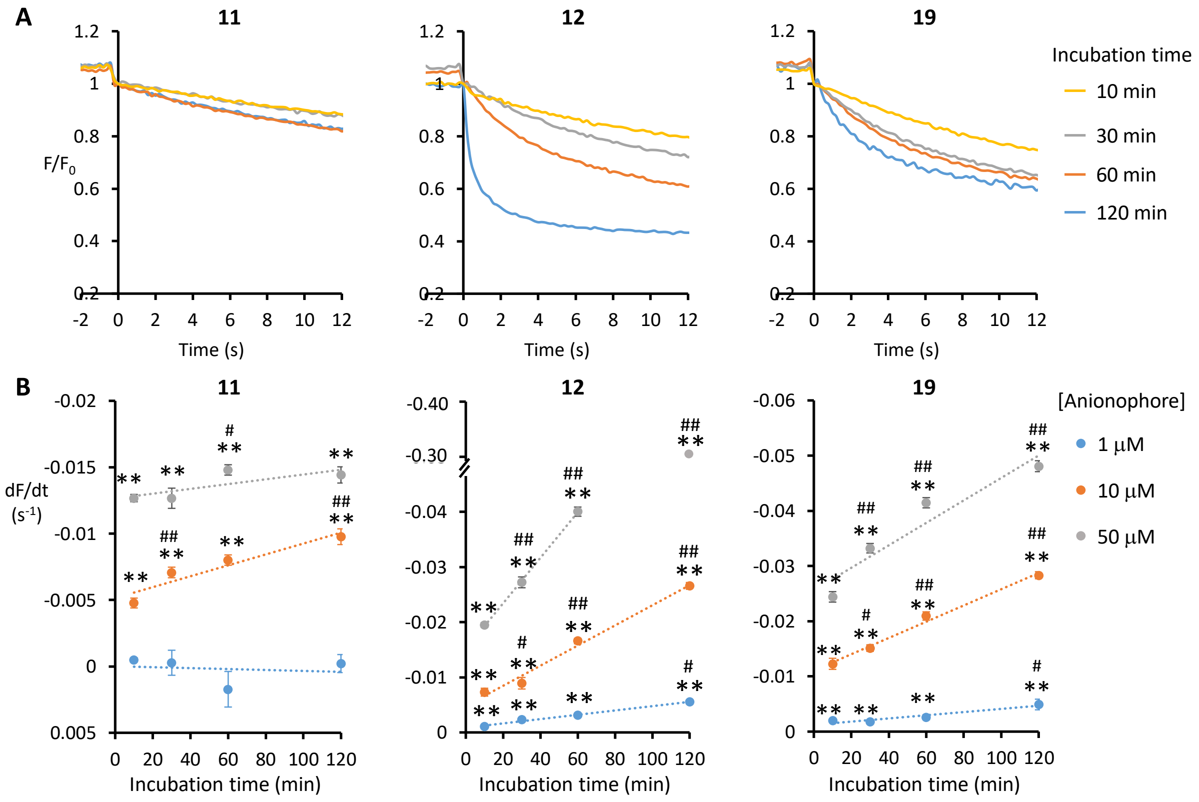

Figure S21. Effects of incubation time on anionophore action. (**A**) Representative time courses of cell fluorescence from YFP-CFBE cells treated with anionophores **11**, **12** and **19** (50 µM) in DMSO (0.5% v v^{-1}) for the specified time intervals before the measurement of cell fluorescence. Cell fluorescence values were normalized to the fluorescence intensity before I^- (100 mM) addition at t = 0 s. (**B**) Concentration- and incubation period-dependence of anionophore-mediated anion transport activity in YFP-CFBE cells determined from the initial slope of the fluorescence decay. Fluorescence quenching by the anionophore vehicle (1 and 10 µM, DMSO 0.1% v v^{-1}; 50 µM, DMSO 0.5% v v^{-1}) was subtracted from test measurement to determine anionophore transport activity. Data are means ± SEM (n = 6 – 21 from at least four independent experiments); $^{\#}$, *P* < 0.05 vs. 10-minute incubation period; $^{\#\#}$, *P* < 0.01 vs. 10-minute incubation period. The dotted lines are the fit of linear regression functions to mean data; note the change in ordinate scale in (**B**).

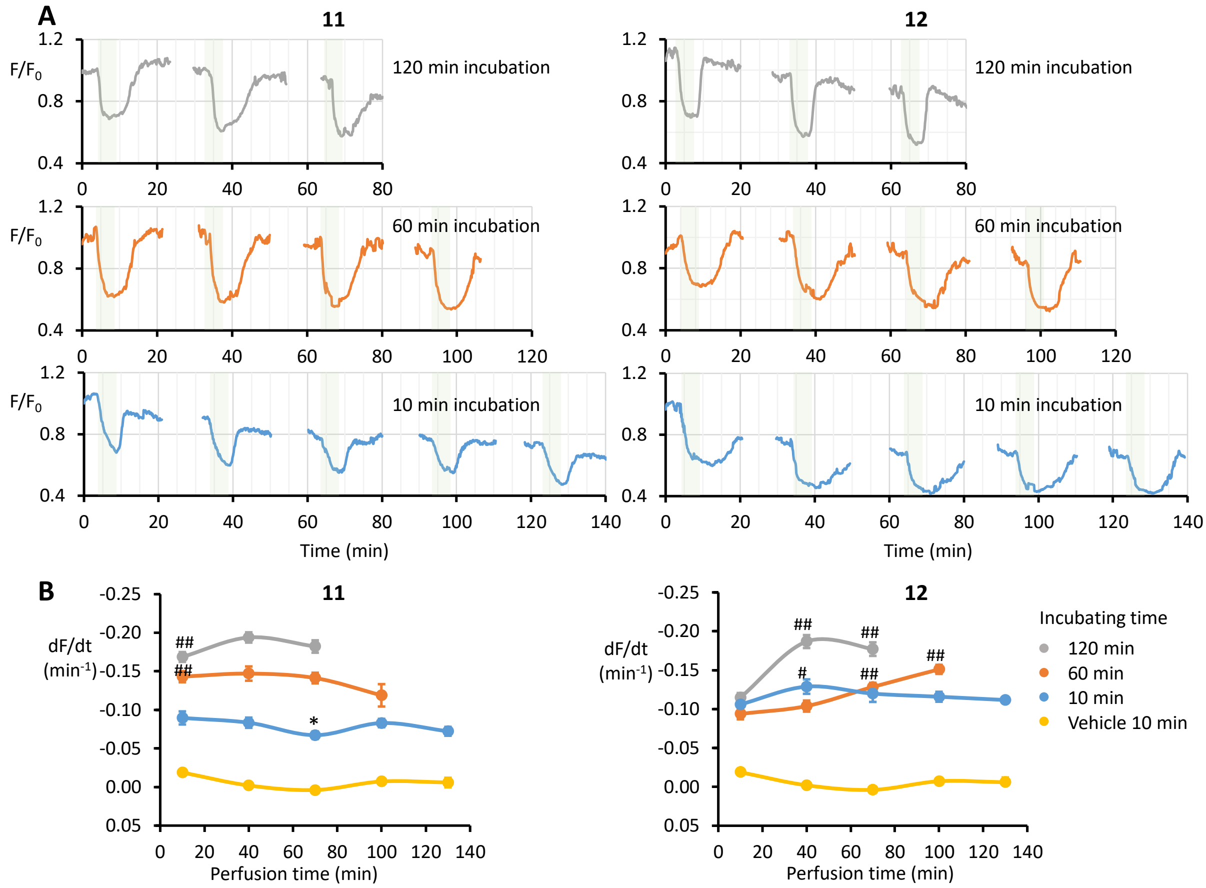

Figure S22. Persistence of anionophore-mediated anion transport. (**A**) Representative long duration time courses of cell fluorescence measured by fluorescence microscopy. YFP-FRT cells treated with **11** and **12** (10 μM) in DMSO (0.1% v v^{-1}) for 10, 60 or 120 minutes were taken through multiple cycles of iodide addition-removal; no data were acquired during the breaks in recording. Cell fluorescence values were normalized to the fluorescence intensity immediately before the first addition of I^- (10 mM). (**B**) Longevity of anionophore activity. Values represent normalized initial rate of fluorescence decay during repeated exposure to I^- (10 mM). Data are means ± SEM (**11**, n = 22 – 37; **12**, n = 28 – 40 from at least 4 independent experiments; DMSO, n = 26 from at least 3 independent experiments); *, $P < 0.05$ vs. 10-minute perfusion of same I^- addition-removal cycle; #, $P < 0.05$ vs. 10-minute incubation of same I^- addition/removal cycle; ##, $P < 0.01$ vs. 10-minute incubation of same I^- addition/removal cycle. In (A), the time course of cell fluorescence for **11** (10-minute incubation) and in (B), the first and fifth data points for **11** (10-minute incubation) and its DMSO control were originally published in ref 10.

Toxicity studies

To evaluate the cytotoxicity of anionophores **11**, **12**, **15** and **19**, we treated YFP-CFBE cells with the anionophores (1 – 50 μM) in DMSO for 18 h at 37 °C before assessing cell viability using sodium 2,3,-bis(2-methoxy-4-nitro-5-sulfophenyl)-5-[(phenylamino)-carbonyl]-2H-tetrazolium) (XTT). Figure S23 summarises the results. Although compounds **12**, **15** and **19** exhibited some cytotoxicity, **11** was without effect at all concentrations tested.

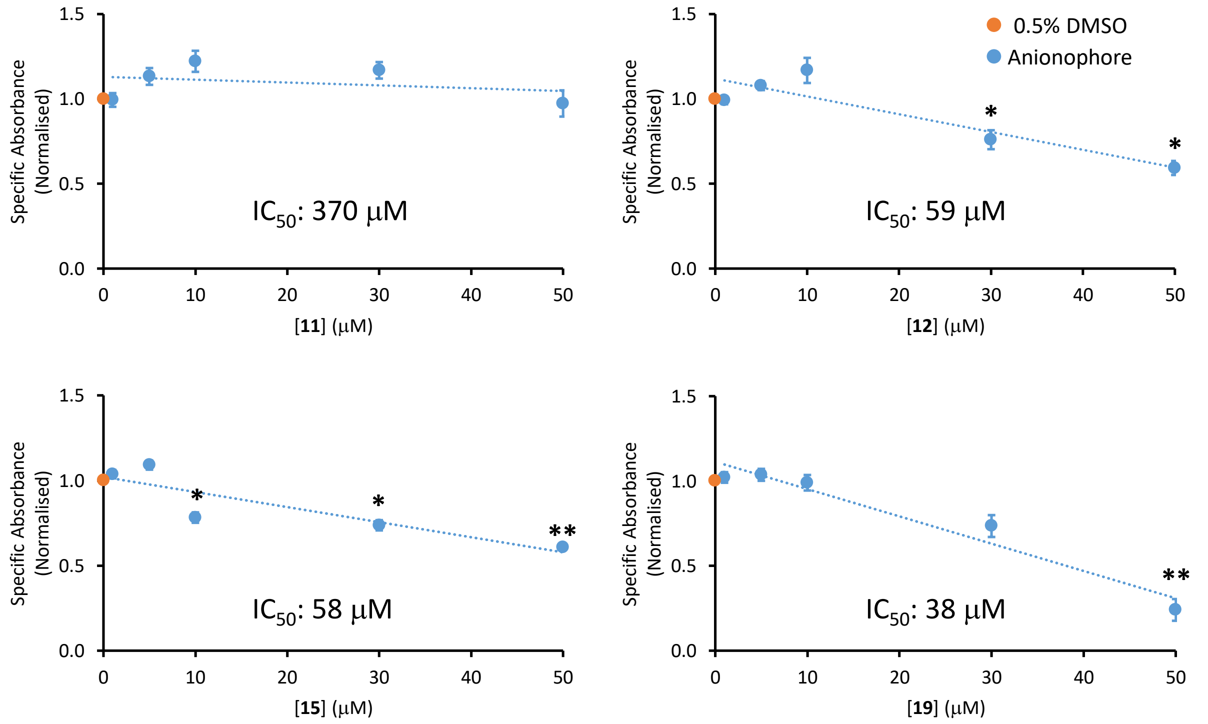

Figure S23. Impact of anionophores on the viability of CFBE cells. CFBE cells were treated with the indicated concentrations of the anionophores **11**, **12**, **15** and **19** or the vehicle (DMSO, 0.5% v v^{-1}) for 18 h before cells treated with XTT and then their viability assessed using XTT. (The total period cells were exposed to anionophores was 22 h). Cell viability is presented as specific absorbance values normalized to that of DMSO (means ± SEM; n = 3 – 5 with 4 replicates per treatment); *, $P < 0.05$ vs. DMSO; **, $P < 0.01$ vs. DMSO). The dotted lines are the fit of first order regression functions to mean data. Values for half-maximal inhibitory concentration (IC_{50}) are indicated for each anionophore.

To investigate further the cytotoxicity of anionophores **12**, **15** and **19** we used YFP-FRT cells. As a control we tested compound **11**. Using the IncuCyte ZOOM™ (Essen Bioscience) live-cell analysis system and the membrane impermeable far-red fluorescent DNA dye DRAQ7 to label the nuclei of dead cells, we examined the effects of the anionophores **11**, **12**, **15** and **19** on the viability of YFP-FRT cells over a 24 h period. Consistent with our previous results (Fig. S23 and ref 10), compound **11** had little effect on cell viability. Only at the highest concentrations tested (30 and 50 μM) and at time points later than 12 h (50 μM) or 14 h (30 μM) did cytotoxicity become apparent ($P < 0.05$ vs. DMSO 0.5% v v^{-1}) (Fig. S24). By contrast, the same concentrations of compounds **12**, **15** and **19** had cytotoxic effects from 2 h with compound **15** (5 and 10 μM) also demonstrating cytotoxicity from 2 h onwards and compound **19** (10 μM) from 8 h onwards ($P < 0.05$ vs. DMSO 0.5% v v^{-1}) (Fig. S24).

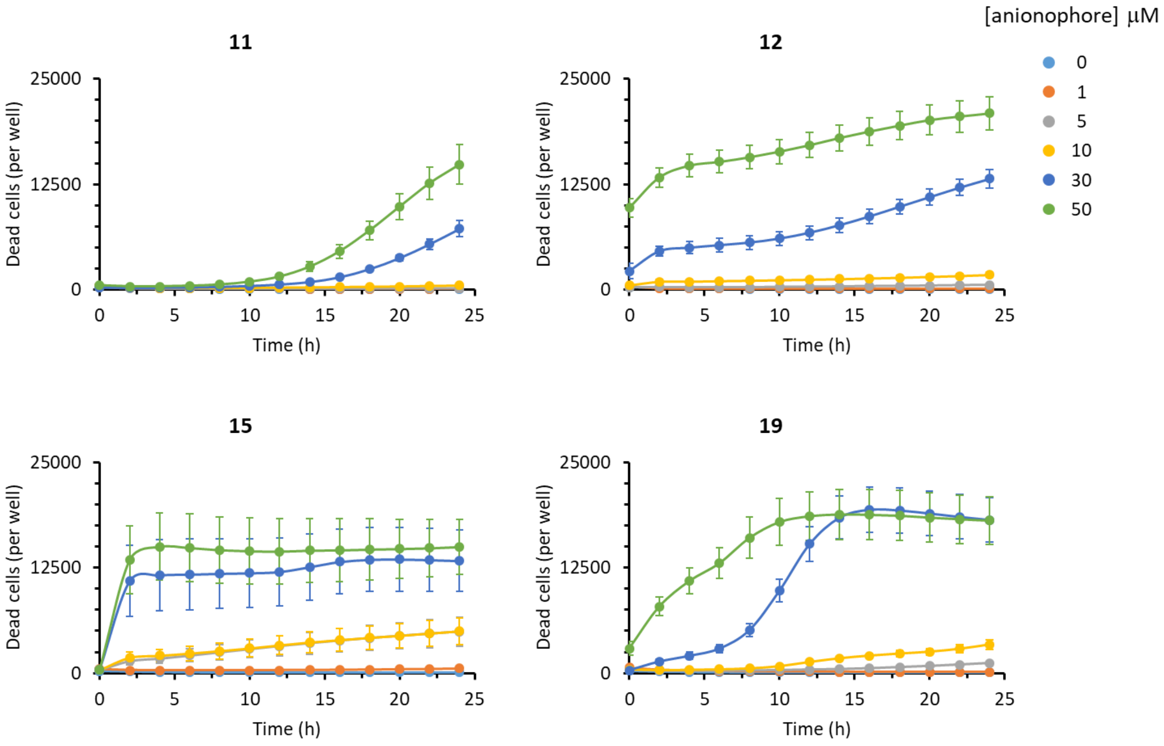

Figure S24. Time- and concentration-dependence of anionophore cytotoxicity. YFP-FRT cells were treated with the indicated concentrations of anionophores **11**, **12**, **15** and **19** or the vehicle (DMSO, 0.5% v v^{-1}) for 24 h. Using the IncuCyte ZOOM™ live-cell analysis system and the far-red fluorescent DNA dye DRAQ7, cell viability was assessed at 2 h intervals for the 24 h period. Data are means ± SEM (n = 5 – 10 with each data point the average of 4 wells).

Previous work has identified that some anionophores trigger apoptotic cell death.[18],[19],[2018,19] Using the IncuCyte ZOOM™ live-cell analysis system and the IncuCyte annexin V red reagent, we assessed whether the anionophores **11**, **12**, **15** and **19** induce apoptosis. Figure S25 demonstrates that compounds **12**, **15** and **19** all trigger apoptosis at concentrations ≥ 10 µM with the effects of 30 and 50 µM apparent from as early as 2 h ($P <$ 0.05 vs. DMSO 0.5% v v^{-1}). However, compound **11** was without effect on apoptosis at all concentrations tested over the 24 h period studied ($P >$ 0.05 vs. DMSO 0.5% v v^{-1}) (Fig. S25). These data confirm our earlier conclusion (ref 10) that the cytotoxicity of anionophores is compound specific and anion transport, by itself, does not trigger cells death. Taken together, the data re-affirm the promising biological activity of anionophore **11**.

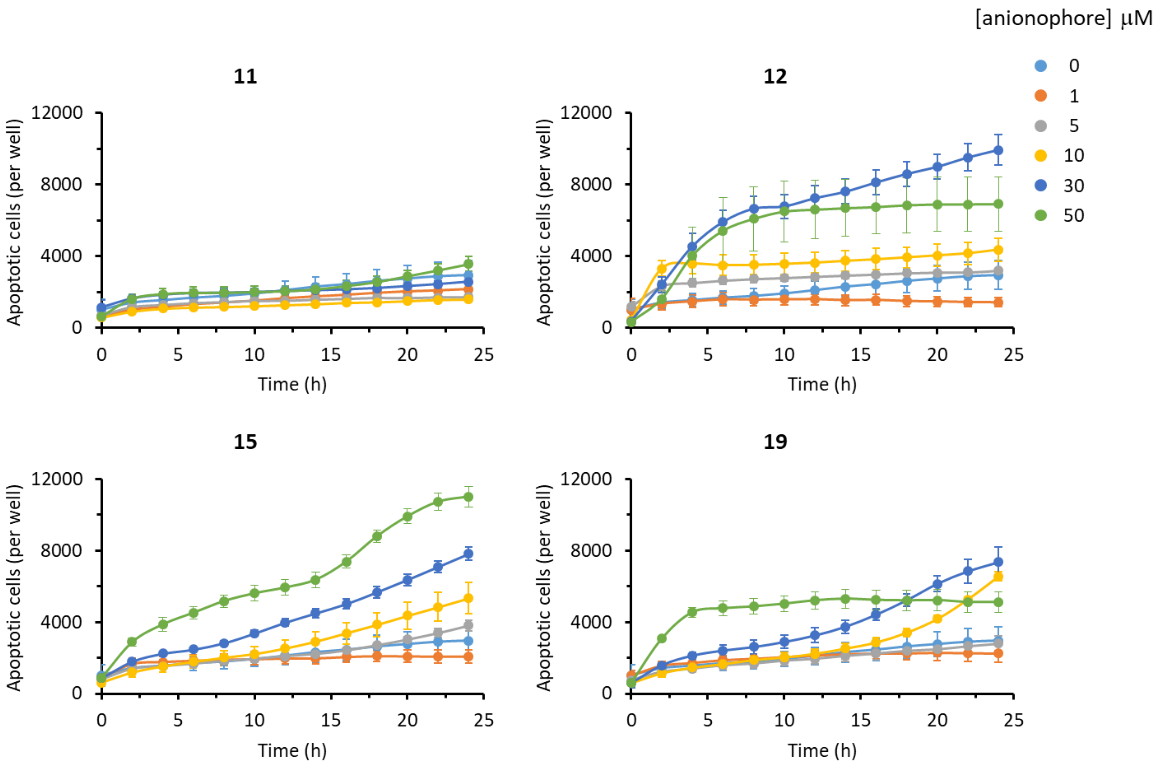

Figure S25. Time- and concentration-dependence of apoptosis induction by anionophores. YFP-FRT cells were treated with the indicated concentrations of anionophores **11**, **12**, **15** and **19** or the vehicle (DMSO, 0.5% v v^{-1}) for 24 h. Using the IncuCyte ZOOM^{TM} live-cell analysis system and the IncuCyte annexin V red reagent, the apoptosis of YFP-FRT cells was determined at 2 h intervals for the 24 h period. Data are means ± SEM (n = 4 with each data point the average of 3 wells).

5. References

1 S. Hussain, P. R. Brotherhood, L. W. Judd and A. P. Davis, *J. Am. Chem. Soc.* 2011, **133**, 1614-1617.

2 H. Valkenier, L. W. Judd, H. Li, S. Hussain, D. N. Sheppard and A. P. Davis, *J. Am. Chem. Soc.* 2014, **136**, 12507-12512.

3 A. B. Pangborn, M. A. Giardello, R. H. Grubbs, R. K. Rosen and F. J. Timmers, *Organometallics* 1996, **15**, 1518-1520.

4 J. P. Clare, A. J. Ayling, J. B. Joos, A. L. Sisson, G. Magro, M. N. Pérez-Payán, T. N. Lambert, R. Shukla, B. D. Smith and A. P. Davis, *J. Am. Chem. Soc.* 2005, **127**, 10739-10746.

5 A. J. Ayling, S. Broderick, J. P. Clare, A. P. Davis, M. N. Pérez-Payán, M. Lahtinen, M. J. Nissinen and K. Rissanen, *Chem. Eur. J.* 2002, **8**, 2197-2203.

6. A. S. Verkman and L. J. V. Galietta, *Nat. Rev. Drug Discov.* 2009, **8**, 153-171.

7. A. Caputo, E. Caci, L. Ferrera, N. Pedemonte, C. Barsanti, E. Sondo, U. Pfeffer, R. Ravazzolo, O. Zegarra-Moran and L. J. V. Galietta, *Science* 2008, **322**, 590-594.

8. D. N. Sheppard, M. R. Carson, L. S. Ostedgaard, G. M. Denning and M. J. Welsh, *Am. J. Physiol.* 1994, **266**, L405-L413.

9. L. V. J. Galietta, S. Jayaraman and A. S. Verkman, *Am. J. Physiol. Cell Physiol.* 2001, **281**, C1734-C1742.

10. H. Li, H. Valkenier, L. W. Judd, P. R. Brotherhood, S. Hussain, J. A. Cooper, O. Jurček, H. A. Sparkes, D. N. Sheppard and A. P. Davis, *Nat. Chem.* 2016, **8**, 24-32.

11. H. Terashima, A. Picollo and A. Accardi, *Proc. Natl. Acad. Sci. USA* 2013, **110**, 19354-19359.

12. A. Schmidt, L. K. Hughes, Z. Cai, F. Mendes, H. Li, D. N. Sheppard and M. D. Amaral, *Br. J. Pharmacol.* 2008, **153**, 1311-1323.

13. W. Namkung, P.-W. Phuan and A. S. Verkman, *J. Biol. Chem.* 2011, **286**, 2365-2374.

14. D. N. Sheppard and M. J. Welsh, *Physiol. Rev.* 1999, **79**, S23-S45.

15. T. Ma, J. R. Thiagarajah, H. Yang, N. D. Sonawane, C. Folli, L. J. V. Galietta and A. S. Verkman, *J. Clin. Invest.* 2002, **110**, 1651-1658.

16. F. Van Goor, S. Hadida, P. D. J. Grootenhuis, B. Burton, J. H. Stack, K. S. Straley, C. J. Decker, M. Miller, J. McCartney, E. R. Olson, J. J. Wine, R. A. Frizzell, M. Ashlock and P. A. Negulescu, *Proc. Natl. Acad. Sci. USA* 2011, **108**, 18843-18848.

17. F. Van Goor, S. Hadida, P. D. J. Grootenhuis, B. Burton, D. Cao, T. Neuberger, A. Turnbull, A. Singh, J. Joubran, A. Hazlewood, J. Zhou, J. McCartney, V. Arumugam, C. Decker, J. Yang, C. Young, E. R. Olson, J. J. Wine, R. A. Frizzell, M. Ashlock and P. Negulescu, *Proc. Natl. Acad. Sci. USA* 2009, **106**, 18825-18830.

18. S. K. Ko, S. K. Kim, A. Share, V. M. Lynch, J. Park, W. Namkung, W. Van Rossom, N. Busschaert, P. A. Gale, J. L. Sessler and I. Shin, *Nature Chem.*, 2014, **6**, 885-892.

19. N. Busschaert, S. H. Park, K. H. Baek, Y. P. Choi, J. Park, E. N. W. Howe, J. R. Hiscock, L. E. Karagiannidis, I. Marques, V. Felix, W. Namkung, J. L. Sessler, P. A. Gale and I. Shin, *Nature Chem.*, 2017, **9**, 667-675.
20. S.-H. Park, S.-H. Park, E. N. W. Howe, J. Y. Hyun, L.-J. Chen, I. Hwang, G. Vargas-Zuñiga, N. Busschaert, P. A. Gale, J. L. Sessler and I. Shin, *Chem*, 2019, **5**, 2079-2098.
