# Supplementary material for: Anion carriers as potential treatments for cystic fibrosis: transport in cystic fibrosis cells, and additivity to channel-targeting drugs
Source: Chem Sci. 2019 Oct 2;10(42):9663–72. doi: 10.1039/c9sc04242c (PMC6984391; doi:10.1039/c9sc04242c)

Electronic Supplementary Material (ESI) for Chemical Science.
This journal is © The Royal Society of Chemistry 2019

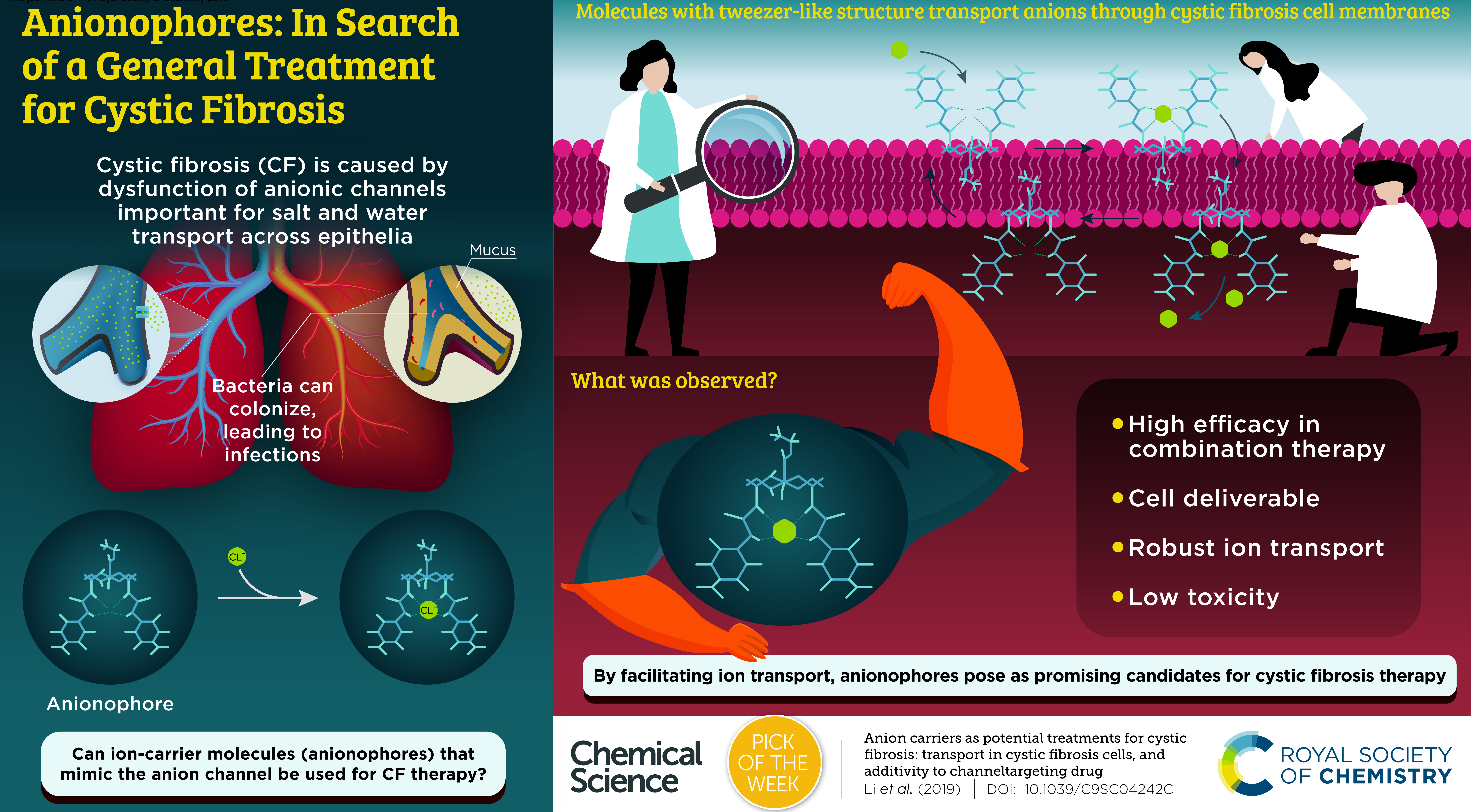

Supplement: Supplementary file 2 [file SC-010-C9SC04242C-s002.pdf]
